# Self-Assembly Versus Surface Charge: Interfacial Response of Ionic Liquids

**DOI:** 10.1021/acs.langmuir.6c03265

**Published:** 2026-09-16

**Authors:** Georgia A. Pilkington, Oliver S. Hammond, Erik Bergendal, Anna Oleshkevych, Stuart J. Brown, Sichao Li, Andrew Nelson, Liliana de Campo, Rebecca Welbourn, Akepati Bhaskar Reddy, Manishkumar R. Shimpi, Sergei Glavatskih, Anja-Verena Mudring, Mark W. Rutland

**Affiliations:** † Division of Surface and Corrosion Science, School of Engineering Sciences in Chemistry, Biotechnology and Health, KTH Royal Institute of Technology, SE-100 44 Stockholm, Sweden; ‡ European Spallation Source, Data Management & Scientific Computing, Asmussens Allé 305, Kongens Lyngby, 2800 Hovedstaden, Denmark; § Department of Materials and Environmental Chemistry, Stockholm University, Stockholm SE-114 18, Sweden; ∥ Department of Biological and Chemical Engineering, Aarhus University, Aarhus N 8200, Denmark; ⊥ School of Chemistry, University of New South Wales, Sydney, NSW 2052, Australia; # Wallenberg Initiative Materials Science for Sustainability, Division of Surface and Corrosion Science, KTH Royal Institute of Technology, SE-100 44 Stockholm, Sweden; ∇ Australian Nuclear Science and Technology Organisation (ANSTO), Lucas Heights, Sydney, NSW 2234, Australia; ○ ISIS Pulsed Neutron and Muon Facility, 120797 Rutherford Appleton Laboratory, Didcot, Oxfordshire OX11 0QX, U.K.; ◆ System and Component Design, Department of Engineering Design, KTH Royal Institute of Technology, SE-100 44 Stockholm, Sweden; ¶ Department of Electromechanical, Systems and Metal Engineering, Ghent University, B-9052 Ghent, Belgium; ⋈ Surfaces, Processes and Formulation, RISE Research Institutes of Sweden, SE-100 44 Stockholm, Sweden; ⧓ Department of Physics, Umeå University, 907 36 Umeå, Sweden; ⧖ Laboratoire de Tribologie et Dynamique Des Systèmes, École Centrale de Lyon, Écully, Lyon 69134, Auvergne-Rhône-Alpes, France; ▼ Chemistry of Interfaces, Luleå University of Technology, Universitetsområdet, Luleå 97187, Norrbotten, Sweden

## Abstract

The bulk and surface charge-driven interfacial structuring of two 1-decyl-3-methylimidazolium (C_10_C_1_Im) orthoborate ionic liquids (ILs), dispersed at various concentrations in the polar aprotic solvent propylene carbonate (PC), were studied using small angle neutron scattering (SANS) and neutron reflectivity (NR). In the bulk, the larger bis­(mandelato)­borate anion reduced the self-assembly capabilities of the blend relative to bis­(oxalate)­borate. At a charged Au electrode interface, a distinct interfacial region was detected in all the IL mixtures studied, which was attributed to two interdigitated bilayers of the cation, containing intercalated anions and solvent, and with bulk IL concentration dependence. Under applied potential, higher concentrations showed clear anion effects on the electro-structuring, whereas dilute solutions were anion independent. The complex bulk and electro-responsive interfacial structuring of such self-assembling IL systems in polar media are pertinent to the application of ILs as designer electrolytes for advanced electrochemical technologies, including next-generation batteries and control over the electrical double layer in capacitors.

## Graphical abstract

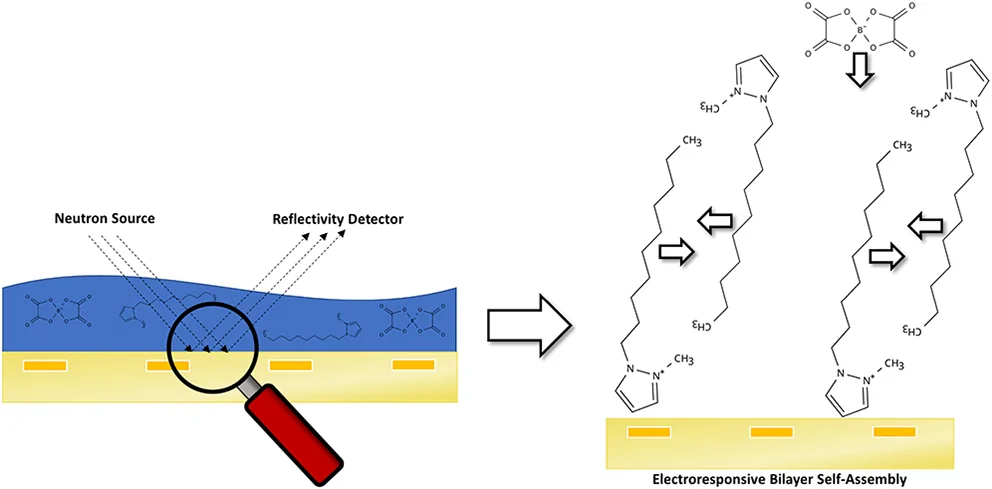

## Introduction

Ionic liquids (ILs) are attractive candidates for energy applications, such as fuel and solar cells, supercapacitors and electrochemical devices, and in tribotronic lubrication, where they have the potential to enhance energy efficiency.^1^ This interest largely stems from the impressive physical properties of ILs, such as low volatility, and generally high thermal and electrochemical stability. The almost limitless combination of ion pairs also makes ILs highly designable, opening the possibility of targeting specific structures and interactions to enhance properties and hence performance. Understanding how the interfacial structuring of ILs at charged surfaces may be controlled is therefore crucial to optimizing their performance. Previous studies have revealed that the electrical double layer structure in ILs is dramatically different to, and more complex than, conventional electrolyte solutions.^2^ Moreover, the interfacial structures of ILs have been shown to be highly sensitive to surface charge, leading to the realization of charge dependent interfacial properties including friction, adhesion, and wetting, largely through controlling the relative ion populations.^3–11^ Other important phenomena, such as mediation of the orientation of heterocycle-bearing ions, have also been reported.^12–14^

Beyond fundamental structuring, there is a clear need for development of new anions for battery/supercapacitor electrolytes. Historically, the majority of commercial ILs contain fluorinated anions (e.g., BF_4_, PF_4_, TFSI), which are thermally unstable and can give rise to hazardous side reactions, leading to safety concerns.^15^ The environmental persistence and bioaccumulation of fluorinated compounds is another issue of concern limiting their usage.^16^ Boron-based chelated orthoborate anions have been identified as particularly promising alternatives for Li-ion batteries, due to their low Li-anode affinities and potential for improving the thermal and cycle stability of supercapacitors, with high power density.^17–19^

The structuring of pure ILs at charged interfaces arises from the interplay of *intra-* (i.e. IL-IL) and *inter-* (ie. IL-surface) -species Coulombic interactions. Depending on the surface charge this can lead to chequerboard, crowding or so-called “overscreening” structures.^2,10^ In some cases, these interactions result in an oscillating (mono)­layer structure, which can extend several ion layers into the bulk.^20^ When “long” alkyl chains are present in one of the ions, the Coulombic structuring is modified by amphiphilic self-assembly of the ions, driven by the preferential segregation of the nonpolar (chains) and the ionic portions into lamellar-like bilayer structures.^21–24^ The critical chain length to induce these effects is system-dependent, varying by ion pair.^21,25^ At electrode interfaces, this unusually long-range ordering of amphiphilic (or surface-active) ILs; SAILs,^26,27^ can translate into significantly higher capacitances at elevated temperatures compared with analogous (mono)­layering IL systems. As a result, bilayer-forming ILs have been suggested as a promising new approach for high-energy-density devices.^28,29^

Neutron reflectometry (NR) is uniquely suited to the characterization of surface-active systems, as it enables direct structural interrogation of solid–liquid interfaces while exploiting isotopic contrast variation to selectively resolve interfacial species. In comparison, many alternative surface-sensitive techniques are limited by significant contributions from the bulk solution, restricted chemical specificity, or practical challenges associated with probing solid–liquid interfaces under operando conditions. When combined with small-angle neutron scattering (SANS), which provides complementary information on self-assembly within the bulk solution,^30^ a comprehensive experimental framework is established for investigating both interfacial and bulk phenomena. These techniques have been widely applied to ionic liquid systems, revealing their propensity for nanoscale self-assembly and their ability to support surfactant aggregation as a function of ion pairing, solvent composition, and ionic liquid-to-solvent ratio.^10,31^

Previously, we have studied a series of imidazolium ILs ([C*_n_* C_1_Im]^+^) of varying alkyl chain lengths, with a nonhalogenated orthoborate anion ([BOB]^−^), at charged interfaces using a combination of sum frequency generation (SFG) spectroscopy and NR.^23^ At sufficient alkyl chain length (*n* = 10), cation bilayers were observed at nonzero surface charge states due to the self-assembly induced disruption of simple electrostatic IL ordering. In addition, for all alkyl chain lengths, and regardless of the extended IL structuring, the planar and charge-delocalized imidazolium ring of the cation was also shown to respond to the surface charge at the electrode interface by modifying its orientation with respect to the surface normal. These observations of bilayer preservation provide a roadmap toward pertinent electrochemical applications, where different surface charge states are surmountable.

In this study, we explore the concentration dependence in the bulk (SANS) and electro-induced interfacial structuring (NR) of two ILs (see Figure 1). The IL pairings use a common 1-decyl-3-methylimidazolium ([C_10_C_1_Im]^+^) cation paired with two different orthoborate anions, bis­(oxalato)­borate ([BOB]^−^) and bis­(mandelato)­borate ([BMB]^−^), which is effectively a diphenylated [BOB]. The *pure* IL [C_10_C_1_Im]­[BOB] is known to form extended bilayers at charged interfaces,^23^ which we extend here to investigate bilayer stability in representative electrolyte solutions. While earlier studies have demonstrated the formation and electrochemical significance of ionic liquid interfacial layers, the present work employs neutron reflectometry to directly resolve the nanoscale interfacial structure under applied electrochemical potential. This study focuses on the self-assembly structuring of these ILs for the future design of IL electrolytes rather than providing a comprehensive assessment of electrochemical performance. The influence of ambient levels of water is also examined, as a frequent unavoidable impurity from synthesis methods and atmospheric exposure, which has previously been shown to lead to dramatic changes in the interfacial properties of IL layers and their electroresponsive ordering.^31–35^

**Figure 1:**
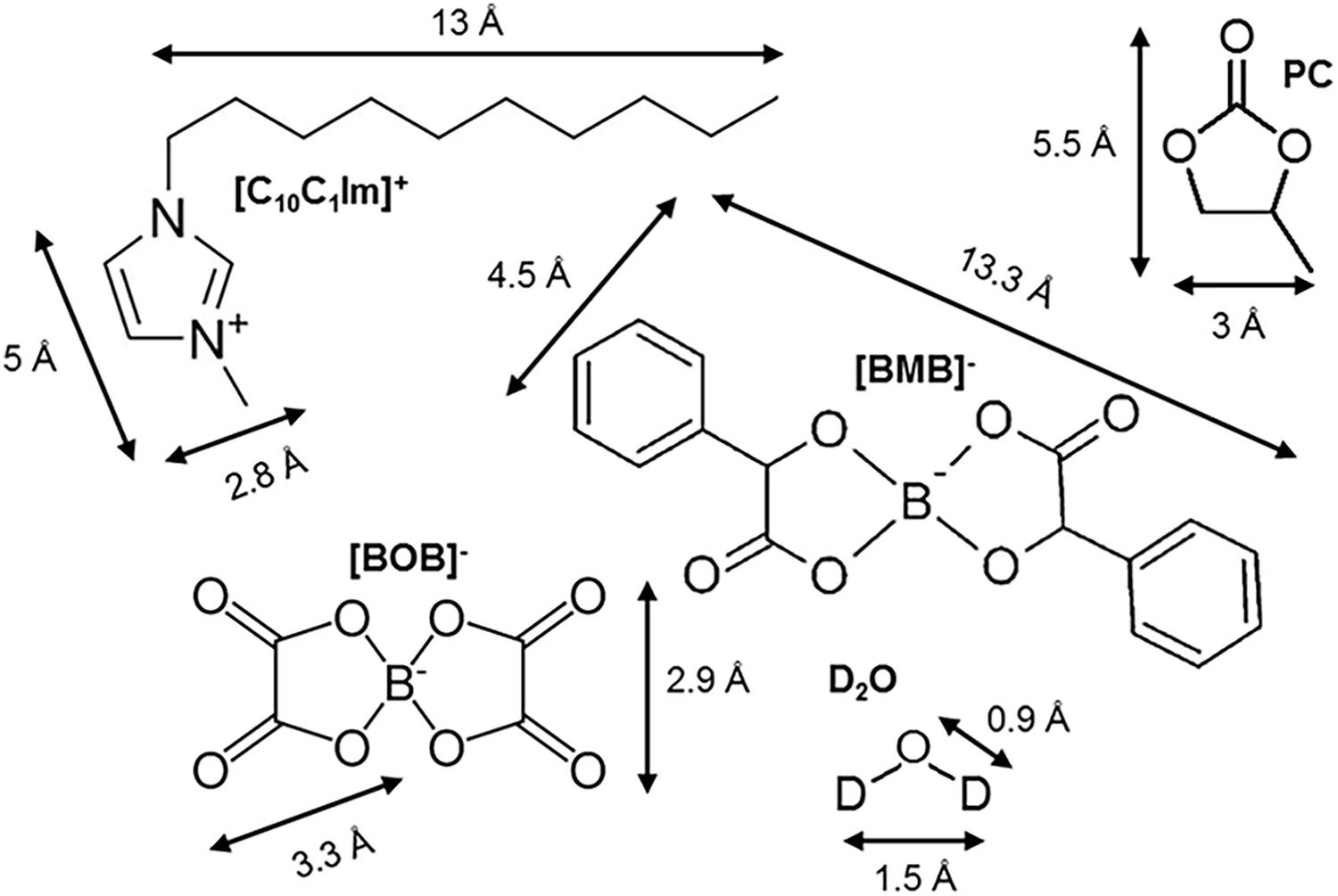
Molecular structures and dimensions of IL ions, the solvent (PC) and D_2_O.

## Experimental Section

### Materials and Solution Preparation

The ILs 1-decyl-3-methylimidazolium bis­(oxalato)-borate [C_10_C_1_Im]­[BOB] and 1-decyl-3-methyl-imidazolium bis­(mandelato)­borate [C_10_C_1_Im]­[BMB] (cf. Figure 1) were provided by Luleå University of Technology. Racemic mandelic acid was used in the synthesis of [BMB]^−^ anions, and thus the [BMB]^−^ anions here can have multiple chiral configurations (i.e., RR, SS, and RS). The effect on physicochemical properties has been discussed by Yue et al.^36^ and Chiappe et al.^37^ We have used the racemic mixture throughout. Future developments in the selective, directed synthesis and isolation of specific stereoisomers would allow further study into how this affects self-assembly behavior.

Prior to all measurements, the ILs were dried in a vacuum oven at 60 °C for approximately 72 h to remove traces of solvents and water. The solvents propylene carbonate (anhydrous, 99.7%; Sigma-Aldrich), 1,2-propylene-*d*_6_ carbonate (99.5 atom % D; Qmx Laboratories Ltd.) and D_2_O (99.9% atom D; Sigma-Aldrich) were used as received. Karl Fischer measurements confirmed low water contents from this preparation procedure, which for [C_10_C_1_Im]­[BOB] was 0.2% w/w. The amount of water in the pure solvent, PC, was determined to be 46 ppm.

For surface measurements, the bulk solution’s scattering length density (SLD) of all solutions was contrast matched to that of gold (see Table 1). The SLDs of the substrate materials is provided in the Supporting Information (SI; cf. Table S1). Ambient water contamination was minimized by preparing the solutions under dry nitrogen (R.H. < 10%) in a glovebag. Solutions were sonicated for at least 10 min until visually homogeneous and either used immediately, or stored under nitrogen. The concentrations of the solutions are presented in terms of IL weight percentage (% w/w) in PC. For NR, three concentrations (5, 20 and 29% w/w) were studied for each IL, representing a dilute (Debye–Huckel), intermediate (20%) and concentrated (29%) solution regime.^38^ 5, 20 and 29% w/w [C_10_C_1_Im]­[BOB] in PC corresponds to 1.29, 5.86, and 9.24 mol %, respectively. 5, 20 and 29% w/w [C_10_C_1_Im]­[BMB] in PC corresponds to 1.00, 4.56, and 7.25 mol %, respectively.

**Table 1:** Molecular Volumes and SLDs of the ILs Studied, Their Individual Ions and Distinct Molecular Portions^a^ ^51^

species	vol /Å^3^	SLD (×10^‑6^ Å^–2^)
[C_10_C_1_Im][BOB]	582	1.53
[C_10_C_1_Im][BMB]	704	1.60
[C_10_C_1_Im]^+^	325	0.33
[C_1_Im]^+^	127	1.79
h-C_10_	201	–0.62
[BOB]^−^	258	3.02
[BMB]^−^	378	2.68
h-PC/d-PC	141	1.53/5.89
D_2_O	30	6.34

a *Estimated from Tanford’s formula.^51^

### Small Angle Neutron Scattering (SANS)

As with NR, samples were prepared from vacuum-dried ILs at a series of concentrations (see Table 2) in *d*_6_-PC in a dry glovebag. After transferring to 1 mm quartz banjo Hellma cells, transmission and scattering measurements were made of the samples using the Bilby SANS instrument at ANSTO,^39,40^ operating in fixed-wavelength mode (as opposed to ToF) with the velocity selector set to provide 4.5 Å incident neutrons (dQ/Q = 10%). Rear and wide-angle detector panels were staggered to cover reciprocal space between 0.0105–0.7212 Å^–1^. Data were reduced in Mantid using calibration measurements of the direct beam, blocked beam, and various sample standards.^41^ Analysis of reduced I­(Q) data was performed by implementing the Teubner–Strey equations in Python and optimizing the curve fits with SciPy.^42,43^ The full fitted parameter set is provided in Table 2, alongside precise experimental sample compositions in various units for convenient reference.

**Table 2:** List of Precise Experimental Sample Compositions for SANS Measurements of IL in *d*_6_-PC Dispersions, Expressed as Mass Fraction, Mole Fraction and Volume Fraction of IL (First Three Columns), Shown Alongside the Full Set of Teubner-Strey Model Fitting Parameters, Namely the Incoherent Background Contribution *c*_0_, *d*-Spacing, Correlation Length **ζ**, and Calculated Amphiphile Strength Parameter **γ**

IL	*w*_IL_	*X*_IL_	Φ_IL_ (actual)	Φ_IL_ (fitted)	*c*_0_ (cm^‑1^)	*d* (Å)	ζ (Å)	γ
C_10_C_1_ImBOB	0.899	0.701	0.905	0.954	0.588 ± 0.005	23.0 ± 0.2	15.8 ± 0.8	–0.90 ± 0.01
	0.704	0.385	0.719	0.768	0.501 ± 0.004	22.5 ± 0.2	11.7 ± 0.6	–0.83 ± 0.02
	0.444	0.174	0.462	0.422	0.306 ± 0.003	21.2 ± 0.2	6.5 ± 0.3	–0.58 ± 0.03
	0.290	0.097	0.305	0.256	0.202 ± 0.002	21.5 ± 0.2	5.3 ± 0.1	–0.41 ± 0.03
	0.200	0.062	0.212	0.205	0.124 ± 0.001	22.1 ± 0.2	4.6 ± 0.1	–0.26 ± 0.03
	0.100	0.028	0.107	0.099	0.068 ± 0.001	24.1 ± 0.2	4.9 ± 0.1	–0.25 ± 0.03
	0.050	0.014	0.054	0.033	0.035 ± 0.000	25.6 ± 0.3	5.0 ± 0.2	–0.21 ± 0.04
C_10_C_1_ImBMB	0.897	0.638	0.897	0.947	0.655 ± 0.002	21.8 ± 0.2	11.1 ± 0.5	–0.82 ± 0.01
	0.699	0.320	0.699	0.749	0.545 ± 0.001	22.6 ± 0.1	6.3 ± 0.1	–0.50 ± 0.01
	0.501	0.169	0.500	0.450	0.395 ± 0.001	26.4 ± 0.1	4.1 ± 0.1	0.02 ± 0.01
	0.290	0.076	0.289	0.239	0.218 ± 0.000	33.1 ± 0.2	3.4 ± 0.0	0.40 ± 0.01
	0.200	0.048	0.200	0.206	0.128 ± 0.001	39.3 ± 1.7	3.0 ± 0.1	0.62 ± 0.03
	0.100	0.022	0.100	0.101	0.076 ± 0.000	34.9 ± 0.3	3.8 ± 0.1	0.37 ± 0.01
	0.050	0.011	0.050	0.048	0.039 ± 0.000	33.5 ± 0.2	4.1 ± 0.1	0.25 ± 0.02

### Neutron Reflectivity (NR)

#### Gold Electrodes

Thin (∼170 Å), amorphous gold films were prepared by electron beam evaporation (Auto 306, Edwards High Vacuum International, Wilmington, MA) atop 10 mm thick (50 × 50 mm^2^) polished silicon (100) blocks (Sil’tronix Silicon Technologies, Archamps, France). To ensure good adhesion between the Si and Au, the Si surfaces were precoated with a thin (∼30 Å) Ti adhesion layer. Prior to the film deposition, the Si surfaces were cleaned in Piranha solution, rinsed with Milli-Q water and dried with filtered N_2_. Before exposing the gold films to the IL solutions, the reflectivity from the coated blocks was first measured in air to characterize the thickness and roughness of the Si, SiO_2_, Ti and Au layers using known values for the SLD of each layer (cf. SI; Figure S1 and Table S1).

Prior to the electrochemical NR measurements, a short length of insulated copper wire was affixed to one corner of each gold surface using a conductive epoxy (CW400, Chemtronics), which was cured in an oven at 120 °C for 20–30 min. The surfaces were then allowed to cool, rinsed with filtered ethanol, blown dry with N_2_ and exposed to UV/ozone for 10 min before mounting in the electrochemical cell.

#### NR Electrochemical Cell

Reflectivity measurements were performed on the IL solutions using a custom-made electrochemical NR cell. Details of the electrochemical NR cell design and its use have been reported previously in related studies of pure ILs and IL-solvent mixtures.^44,45^ Therefore, a detailed description of the NR cell and methodology is confined to the SI. Briefly, a gold working electrode and fluorine-doped tin oxide conductive glass counter electrode were used in a potential range of −1.5 to 0.25 V (using either a Metrohm Autolab 302 or PGSTAT204 potentiostat at ANSTO or ISIS respectively). The potential was limited to +0.25 V to avoid electrostripping of the gold,^44^ while each potential was applied for 30 min prior to NR measurements to allow for the cell to reach an equilibrium state. This allows for continued experimentation after an applied +0.25 V and ensures reproducible measurements. Surface charge density of the gold electrode was calculated by integrating the observed electric current (cf. Figures S2 and S11), assumedly due to ion diffusion.^46^ The surface area of the working electrode was defined by a Teflon gasket and estimated to be 17.6 cm^2^. Post reflectivity cyclic voltammetry (CV) measurements were conducted in the potential range for all ILs to identify potential Faradaic events and identify reversibility of the system.^44^ These were conducted at a scan rate of 10 mV/s repeating several times (cf. Figures S3 and S12).

#### NR Measurements

The specular neutron reflectivity, R, was measured as a function of the momentum transfer vector, *Q*_*z*_ = 4π sin θ/λ, where θ is the angle of incidence and λ is the wavelength of the neutron beam. The neutron beam was directed through the Si block and reflected from the gold-IL solution interface, with the reflected beam again traversing the block and exiting to the detector. In all cases the NR measurements were conducted with the working electrode in a horizontal geometry.

The NR measurements presented here for 5 and 29% w/w [C_10_C_1_Im]­[BOB] and 5, 20 and 29% w/w [C_10_C_1_Im]­[BMB] (in contrast matched PC) were performed using the PLATYPUS time-of-flight reflectometer^47^ at the 20 MW OPAL reactor (ANSTO, Sydney, Australia) at ambient temperature. The NR measurements for 20% w/w [C_10_C_1_Im]­[BOB], with and without added D_2_O in contrast matched PC, were performed on the INTER time-of-flight reflectometer at the ISIS Facility, Rutherford Appleton Laboratory, U.K..^48^ Further reflectivity instrument details are presented in the Supporting Information.

#### NR Analysis

The SLD profiles were obtained from best-fits to the NR data performed using the software package GenX and a logarithmic figure of merit (FOM).^49^ The data were fit using a slab model based on the reflections from a stratified medium; i.e., to a model consisting of a series of layers, each with an associated thickness, roughness and scattering length density (SLD). As the gold interfacial roughness (∼10 Å) was found to be comparable to the molecular dimensions of the IL (cf. Figure 1), the usual Nevot-Croce approach^50^ to the roughness was deemed unsuitable at this interface. Instead, the gold-IL interface was modeled using a microslabbing approach (defined in Supporting Information), which has been previously implemented for other IL solutions.^31^ This parameter, along with the coupled parameter of the Au thickness, and the bulk IL solution SLD were fitted for the 0 V and fixed for all subsequent potential conditions. All other parameters related to the substrate (i.e., the Ti and SiO_2_ thickness, roughness and SLD), were fixed to those obtained from the substrate reflectivity measurements in air. The thickness, roughness and SLD values of the IL layers were allowed to vary between potentials.

Best fits to the reflectivity data were determined by the lowest figure of merit (FOM) for a minimum number of layers. The SLD of the bulk solution was also kept constant for the different potential conditions studied. Initial fits were performed for a one-layer model and subsequent layers added if the FOM value decreased. The simulations were performed independently and no predefined structures were assumed. For all the fitted data sets, the thickness, roughness and SLD of each of the interfacial layers, as well as the roughness of the gold/IL and IL/bulk interfaces were allowed to vary. The thickness of the interfacial region was limited to a minimum value of the gold roughness measured in air. The layer SLDs were constrained to be within the range of the possible component species. The estimated SLD of the pure ILs were calculated from their measured mass densities ([C_10_C_1_Im]­[BOB] = 1.17 g cm^–3^; [C_10_C_1_Im]­[BMB] = 1.26 g cm^–3^). The SLDs of the ions and their molecular portions were estimated from calculated molecular volumes (cf. Table 1). The SLD of a molecule is the sum of the SLDs of its components multiplied by their respective volume contributions to the molecule. It is to be noted that due to the presence of several molecular species in the IL mixtures, the exact composition of the layers cannot be extracted from the fitted layer SLDs presented and even extensive calibration data would not remove this ambiguity.

## Results and Discussion

### Bulk Nanostructure of [C_10_C_1_Im]­[BOB] and [C_10_C_1_Im]­[BMB] in PC: Effect of Concentration

The nanoscale organization in bulk dispersions of [C_10_C_1_Im]­[BOB] and [C_10_C_1_Im]­[BMB] in *d*_6_-PC was investigated using SANS (Figure 2). In this case, contrast matching was not performed and instead, the “ideal” scattering case with maximum contrast and minimum incoherent background contribution was sought. Both IL data sets show an I­(Q) decay and mid-Q “peak” characteristic of two-component microemulsion-type systems, and thus ideal candidates for fitting using the shape-independent Teubner–Strey (TS) model.^42,52,53^

**Figure 2:**
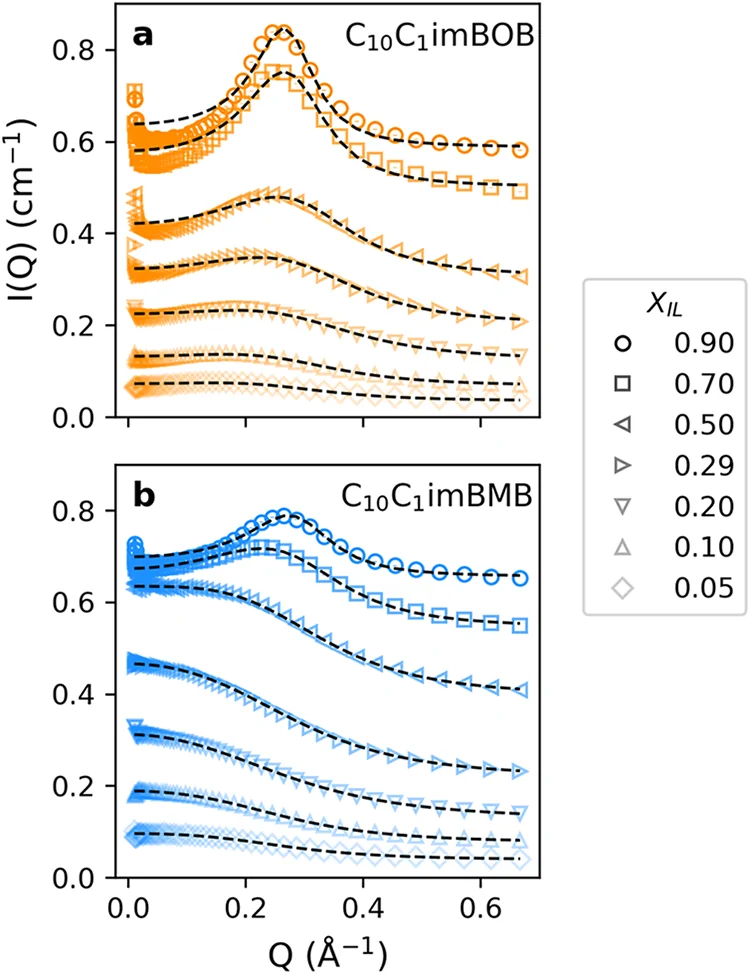
SANS data (markers) for dispersions of [C_10_C_1_Im]­[BOB] (a; top) and [C_10_C_1_Im]­[BMB] (b; bottom) in *d*_6_-propylene carbonate measured using BILBY. Data are normalized to an absolute intensity scale and represent samples with sequentially higher IL concentration as the scattered intensity increases; the markers correspond to IL mass fractions of ca. 0.05 (diamonds), 0.1 (triangles), 0.2 (down-pointing triangles), 0.29 (right-pointing triangles), 0.5 (left-pointing triangles), 0.7 (squares) and 0.9 (circles). Fits to each data set using the Teubner–Strey model are plotted as black dashed lines.

Clear distinctions between the I­(Q) for [BOB] and [BMB] ILs can be seen with the reduced SANS data plotted alongside the corresponding model fits (Figure 2). Qualitatively, the mid-Q peak intensity in the [BMB] case is smaller, and the peak intensifies for both anions with increasing IL concentration. The data indicates that the more hydrophobic anion (BMB) and/or lower concentrations have less well-defined self-assembly structures. A comprehensive list of the fitted TS model parameters is given in Table 2 as well as the mass, mole, and volume fraction of IL in each experimental sample.

Despite being morphology agnostic, the empirical fitting parameters in the TS model allow us to fully quantify the self-assembly characteristics of these systems. These parameters include the *d*-spacing, correlation length ζ, and from these the calculated amphiphile strength parameter γ, which has been described previously.^54^ The variation in these parameters is shown as a function of IL mole fraction in Figure 3. Figure 3a also shows the “Lifshitz line” (where γ = 0), which indicates the transition from weakly structured mixture to spongelike bicontinuous (“microemulsion-type”) structuring.^52^ For the pertinent spongelike case where −1 ≤ γ ≤ 0, the *d*-spacing corresponds to the width of the alkyl chain domain in the solvophobically driven self-assembly structure, and the correlation length ζ provides information on the mean extent of these transient regions of bicontinuous assembly (ie. proportional to aggregation number^54,55^ ). To avoid confusion, we will not delve into the alternate meaning(s) of these parameters beyond the phase transition where 0 ≤ γ ≤ 1, at which point the system is categorized as weakly structured.

**Figure 3:**
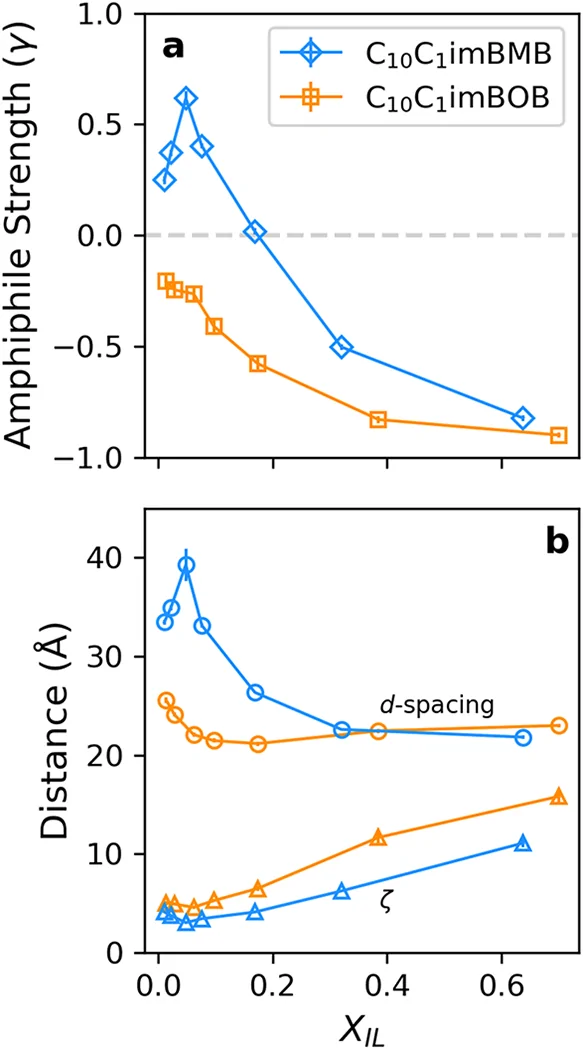
Fitted parameters for [C_10_C_1_Im]­[BOB] (blue markers) and [C_10_C_1_Im]­[BMB] (orange markers). (a) The calculated amphiphile strength parameter, **γ**, as a function of IL mole fraction. The gray dashed line drawn at **γ = 0** is the Lifshitz line. (b) The fitted *d*-spacing (circles) and correlation length, **ζ** (triangles) values obtained from the Teubner–Strey model fits.

The key finding of these SANS experiments is therefore shown in Figure 3a and is namely that the amphiphile strength parameter for [C_10_C_1_Im]­[BOB] is always negative, making this a strong spongelike assembly former at all tested concentrations of IL in PC. Conversely, this is only the case at rather high mole fractions of [C_10_C_1_Im]­[BMB], where spongelike behavior is observed only for >50 wt % IL in PC. When both systems are in this self-assembly regime, the *d*-spacing will be quite similar and within the experimental error of SANS. This is expected behavior considering that this aspect is largely defined by the C_10_ chain. However, a major difference is found in the correlation length ζ, which is universally higher for [C_10_C_1_Im]­[BOB] and indicates a greater mean aggregation number. We are therefore able to conclude from this bulk information that the lower polarity (more hydrophobic) [BMB] anion, with its appended phenyl groups, disrupts the [C_10_C_1_Im] self-assembly. An analogous behavior has been observed previously in SANS measurements of the pure bulk ILs, where it was hypothesized that the larger anion size increased the bulk percolation threshold through topological effects, and the lower polarity reduced the thermodynamic driving force for entropically driven self-assembly.^30^ This understanding of the bulk defines a framework for understanding the more complex interfacial electro-responsive structuring behavior below.

### Electro-Driven Interfacial Structuring of [C_10_C_1_Im]­[BOB] in PC: Effect of Concentration

The reflectivities of three different concentrations of the IL [C_10_C_1_Im]­[BOB] in PC are shown as a function of applied potential at a gold electrode interface in Figure 4. For all concentrations and potential conditions, clear Kiessig fringes are observed in the reflectivities. Since the bulk solution is contrast matched to that of gold, (considering the IL contribution to the bulk SLD) the fringes can only originate from a thin, (absorbed) IL rich film at the Au electrode, of rather different SLD to that of the bulk. The fringes appear to have the same spatial frequency as that of the gold because the IL layer is separated from the next *step-change* in SLD (the TiO_2_ layer) by the gold layer thickness. If there were no interfacial film present, then a featureless reflectivity profile would be produced.

**Figure 4:**
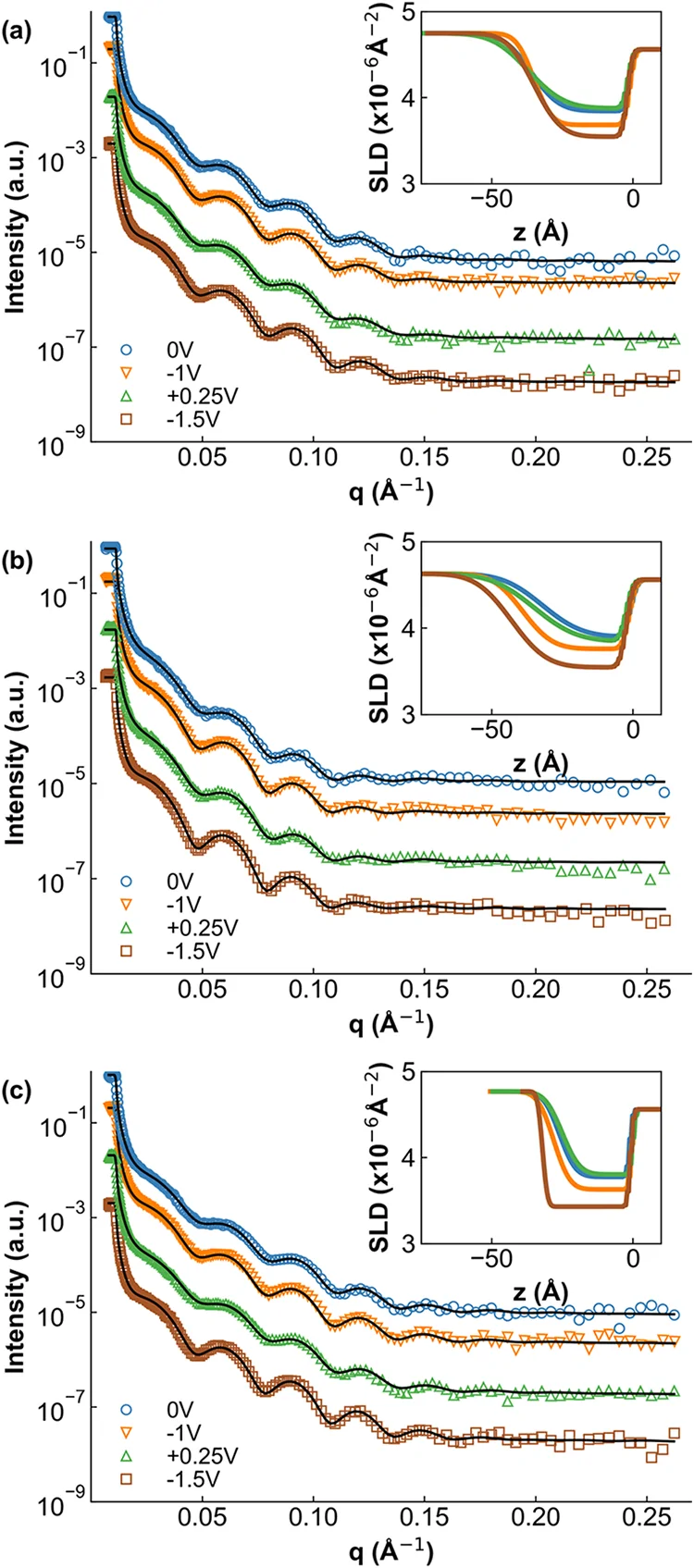
Reflectivity measured for a solution of (a) 5, (b) 20 and (c) 29% w/w [C_10_C_1_Im]­[BOB] at different applied potentials at a gold electrode interface. The symbols represent the experimental data, while the solid lines represent best-fits to the data. The insets show SLD profiles for different applied potentials obtained from the best-fits presented, where the gold-IL interface is located at *z* = 0. The potentials are presented in chronological ordering of the NR experiment from top to bottom.

With applied potential, variation in amplitudes of the Kiessig fringes indicate changes in the SLD of the near-surface region. Asymmetry plots showing the relative magnitudes of these changes with respect to the reflectivities initially measured at 0 V can be found in the SI (cf. Figure S4). With increasing bulk IL concentration, the disparity between the reflectivities measured for different surface potentials is more evident.

For all studied potential conditions and bulk IL concentrations, a 1-layer SLD model was sufficient to accurately capture the features in the measured reflectivities. Best fits to each data set are shown in Figure 4 (solid lines). Corresponding SLD profiles as a function of distance from the gold interface (*z* = 0) are shown in the figure insets. The SLD profiles reveal distinct changes in the interfacial thickness and average SLD of the interfacial region with applied potential, which also depend on the bulk IL concentration. The fit parameters corresponding to these SLD profiles are given in Table S2 in the SI.

Previously, NR and vibrational sum frequency spectroscopy (VSFS) measurements demonstrated responsive self-assembly structuring of the same IL in its pure form at charged interfaces.^23^ Depending on the surface potential, the overall thickness of the bilayer (including a counterion layer) in the pure IL was determined to be ∼20–23 Å. SANS measurements in the same (pure) IL similarly revealed a sponge phase, composed of continually connected disordered bilayers, with a *d*-spacing of 23 Å.^30^

Here, the presence of solvent smears the signal (rendering a single layer formally sufficient to fit the data).^23^ If, however, the number of fitting layers is permitted to increase, then some variations are apparent in the IL SLD profiles (cf. Figures S6a and S7a). Moreover, these variations are consistent with those expected for an interfacial region composed of two intercalated cation bilayers. However, the specific packing of the cations in these layers and coexistent anion concentration normal to the interface seem to vary depending on the bulk IL concentration and applied potential. With a 1-layer fit, we observe the convolution of these changes.

At 5% w/w, the thickness of the interfacial region increases slightly toward the positive potential (cf. Figure 4a **inset**). The magnitude of this thickness change is within the error of these measurements, but also comparable to the expected thickness change (cf. SI, Table S2) for reorientation of the imidazolium ring, which has been previously shown to occur at different surface potentials on gold in the pure IL.^14^ More specifically, the imidazolium ring is expected to adopt a more parallel orientation relative to the surface at negative potentials, leading to a small thickness decrease, while at a positive surface potential the ring adopts a perpendicular orientation, increasing the layer thickness.^13,14,23^

Upon increasing the bulk IL concentration to 20% w/w, more significant changes in the layer thickness occur upon changing the surface potential. At nonzero potentials, the layer becomes thicker (cf. Figure 4b **inset**), which is consistent with observations from previous electrochemical NR measurements on pure IL, [C_10_C_1_Im]­[BOB].^23^ Such thickness variations were largely attributed to changes in the density and degree of ordering of the cations in the first layer.

At a larger negative surface potential (−1.5 V), the surface adjacent bilayer is accompanied by a similar, but more diffuse (high roughness), bilayer-like structure atop it, indicating that the surface charge is too high to be compensated by a single counterion layer. At a positive potential (+0.25 V) the observed increase in the thickness of the interfacial region as compared to 5% w/w, was also correlated to an increased population of anions as a concentration effect, inferred from the higher SLD.

A further increase of the IL concentration, to 29% w/w, reveals a similar trend, although the interfacial region is thinner overall (cf. Figure 4c **inset**) and comparable with that of the bicontinuous phase thickness (*d*-spacing) measured in the pure IL by SANS.^30^ The increased thickness of the interfacial region with applied potential at these higher concentrations points toward a reduced ability of the ions to respond to the changes in surface potential via a simple ion exchange mechanism as compared to the lower potential and lower concentration solutions. This is probably due to the lower solvent content and concomitantly reduced screening between the ion pairs at the interface. Instead, the ions compensate the surface charge via formation of multilayers, resulting in a thickening of the interfacial region in a phenomenon commonly referred to as overscreening (cf. Figure 5).^2^ Briefly, the self-assembly properties conferred by the hydrocarbon chains lead to a bilayer type structure on the surface (the density of which depends on the surface potential). This leads to two planes of cationic charge, one close to the surface and one further out. This association results in the positive charge compensating the surface charge, which then necessitates a further layer of anions to permit the orthogonal constraints of self-assembly and electroneutrality to be met. The same arguments have been made for SAILs, where the anions of an ionic liquid system are capable of both analogous self-assembly and the formation of alternating charged layers.^28,29,56^ Precluded by limited experimental beamtime, one potential avenue of future study would be to investigate whether the balance of intermolecular interactions is significantly altered by temperatures beyond room temperature, and within the operating window for real electrochemical devices.

**Figure 5:**
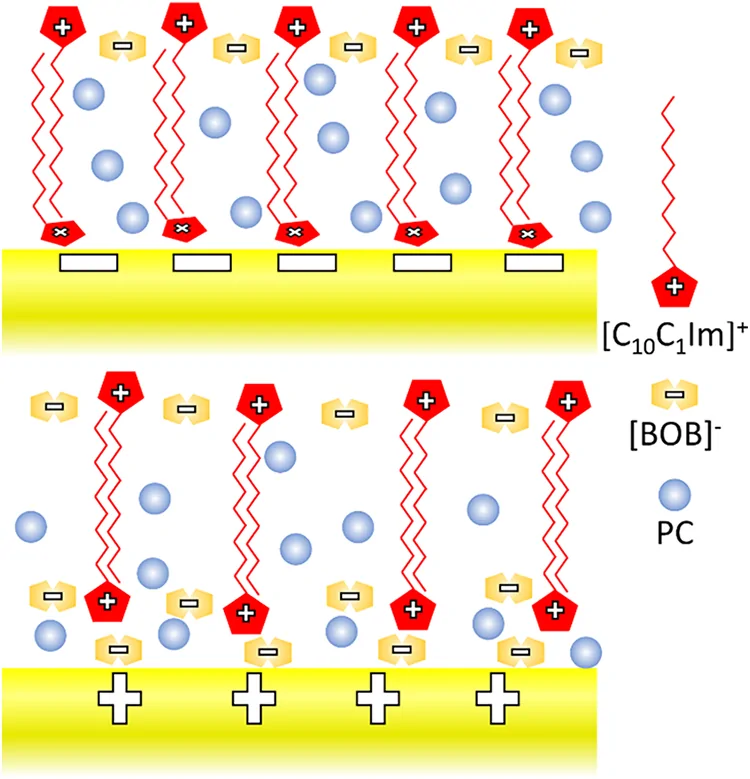
Visual representation of the bilayer structure observed at nonzero potential for [C_10_C_1_Im]­[BOB] at 29% w/w. At negative potential it notes the parallel orientation of the imidazolium ring to the electrode surface while at both potentials’ intercalation of anions and cation alkyl chains are key components of the bilayer.

Between the different IL concentrations, the SLD of the interfacial region does not vary significantly (cf. Figure 4 **insets;** SI, Table S2), but the lower SLD for 29% w/w at all potentials is consistent with a more densely packed layer (i.e., lower solvent fraction). With applied potential, a general increase in the layer SLD toward the positive potential is also observed for all concentrations of the IL. This indicates a higher fraction of the higher SLD anions (cf. Table 1) at the interface under these conditions, assuming a constant solvent fraction at the interface. The validity of this assumption cannot be confirmed directly; related measurements using QCM to study changes in the surface composition with potential at similar compositions suggest that only ion exchange takes place in the interfacial films in response to the potential.^57^

### Electroresponsive Structuring of [C_10_C_1_Im]­[BMB] in PC: Effect of Concentration

The reflectivities for three different concentrations of the IL [C_10_C_1_Im]­[BMB] in PC as a function of surface potential at a gold electrode surface are shown in Figure 6. As for the solutions of [C_10_C_1_Im]­[BOB] in PC, Kiessig fringes, which originate from a solvated layer of IL next to the gold electrode, are present for each bulk concentration and vary with applied potential. However, these changes are generally not as significant. Asymmetry plots showing the relative magnitudes of these changes with respect to the reflectivities initially measured at 0 V can be found in the SI (cf. Figure S5). With increasing bulk IL concentration, it is evident that the disparity between the reflectivities measured for different surface biases increases. However, in the asymmetry plots for the negative biases of −1 and −1.5 V the reflectivities are notably more similar in magnitude than those observed for the [BOB]^−^ solutions.

**Figure 6:**
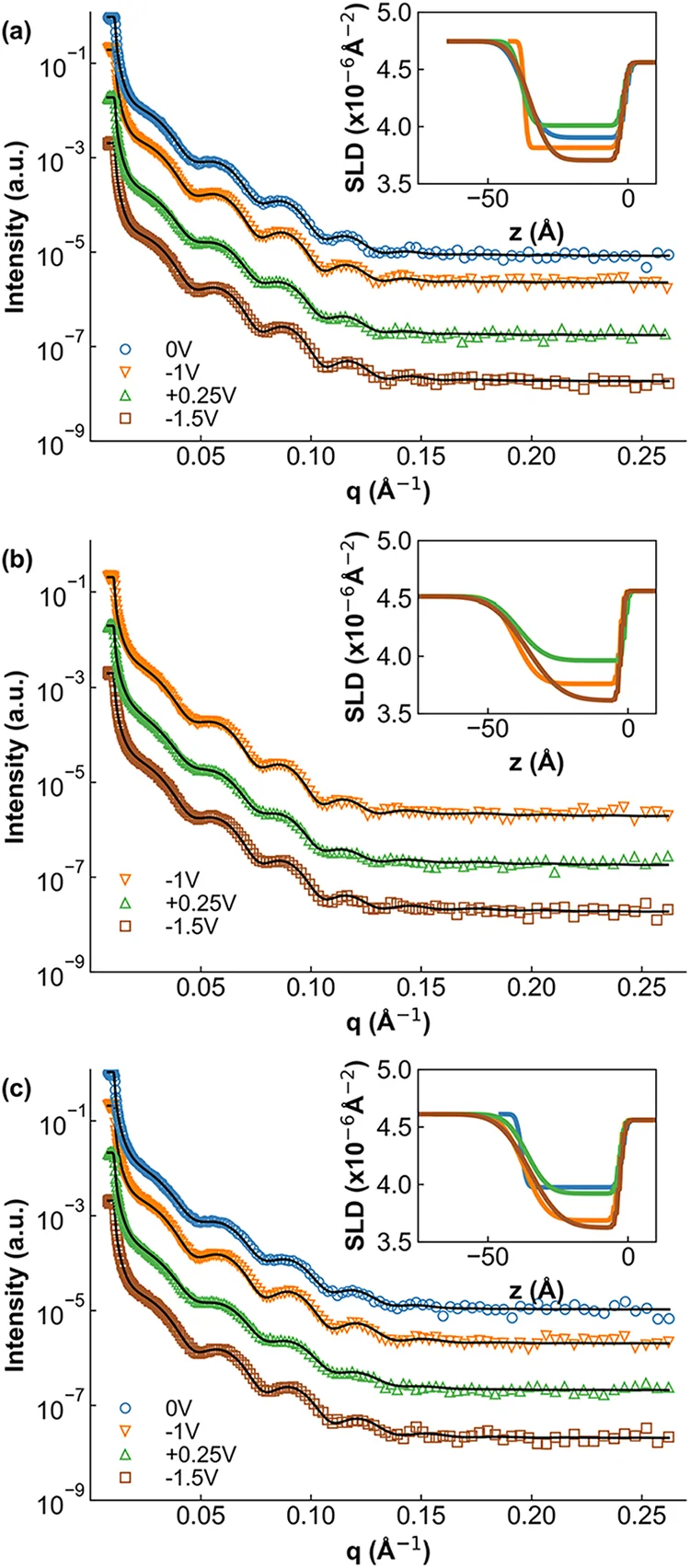
Reflectivity measured for a solution of (a) 5, (b) 20 and (c) 29% w/w [C_10_C_1_Im]­[BMB] at different applied potentials at a gold electrode interface. The symbols represent the experimental data, while the solid lines represent best-fits to the data. The insets show SLD profiles for different applied potentials obtained from the best-fits presented, where the gold-IL interface is located at *z* = 0. The potentials are presented in chronological ordering of the NR experiment from top to bottom.

Similar to the [C_10_C_1_Im]­[BOB] solutions, a 1-layer SLD model was sufficient to capture the features of the reflectivities for all conditions and bulk IL concentrations. Best fits to each data set are shown in Figure 6 (solid lines). Corresponding SLD profiles as a function of distance from the gold interface (*z* = 0) are shown in the figure insets. Compared to the solutions of [C_10_C_1_Im]­[BOB] in PC, the interfacial structuring of [C_10_C_1_Im]­[BMB] exhibits a weaker dependence on the IL bulk concentration.

In contrast to the [C_10_C_1_Im]­[BOB] solutions, the thickness of the interfacial region does not appear to change significantly between 5 and 29% w/w. Instead, a general increase in the layer thickness of the interfacial region from negative to positive potentials is observed, for all bulk IL concentrations (cf. Figure 6 **insets;** SI, Table S2). (Note that due to experimental beamtime exigencies, the reflectivity for 20% w/w­[C_10_C_1_Im]­[BMB] at 0 V was not measured.) Moreover, the layer thicknesses toward the positive potential are notably larger than measured for the [C_10_C_1_Im]­[BOB] solutions. Both of these trends can be attributed to the larger molecular volume and size of the [BMB]^−^ anion (258 Å^3^ and 378 Å^3^, cf. Table 1 and Figure 1, respectively). Concomitantly, the layer SLD also increases toward the positive potential (cf. Figure 5 **inserts**), consistent with a larger, high SLD anion fraction at the interface (cf. Table 1). It is also noticeable that the layer SLDs for the [C_10_C_1_Im]­[BMB] solutions are generally higher, despite the lower theoretical SLD of the [BMB]^−^ anion (cf. Table 1).

Previously, it has been reported that the [BMB]^−^ is expected to pack less efficiently,^31^ leaving more interstices for the solvent (which would lead to a global increase in the layer SLD). This less efficient packing of the anions at the interface is also likely to contribute to the larger layer thicknesses measured toward the positive potential. In contrast, at the most negative potential (−1.5 V) the layer thickness was comparable to that measured for [C_10_C_1_Im]­[BOB], i.e., when the structuring is expected to be largely determined by the larger fraction of [C_10_C_1_Im]^+^ cations. Another significant difference caused by the anions at 20 and 29% w/w [C_10_C_1_Im]­[BOB], is that the IL layer at negative potential is much thicker than at positive potential, the opposite of what is observed for [C_10_C_1_Im]­[BMB]. However, unlike the case for the [BOB]^−^ anions (cf. Figure 3), no changes in the interfacial region thickness with a change from −1 V to −1.5 V were observed (cf. Figure 4).

This finding is consistent with molecular dynamics (MD) studies on IL/electrode interfaces that suggest that the larger IL ions exhibit a greater tendency toward ion-crowding behavior^58^ and implies that the larger size of the [BMB]^−^ leads to the exclusion of the anions from the cationic bilayer region (cf. Figure 7). Moreover, recent SANS measurements of these two (pure) ILs revealed that [C_10_C_1_Im]­[BMB], similar to (pure) [C_10_C_1_Im]­[BOB], forms a sponge phase composed of continually connected disordered bilayers, but with a lower aggregation number and hence inferior self-assembly capability as represented by the amphiphile strength parameter, implying disruption of the solvophobic segregation by the [BMB]^−^ anion.^30^ Speculatively, π-π interactions between the [BMB]^−^ phenyl rings and cation imidazolium rings could also play a contributing role.

**Figure 7:**
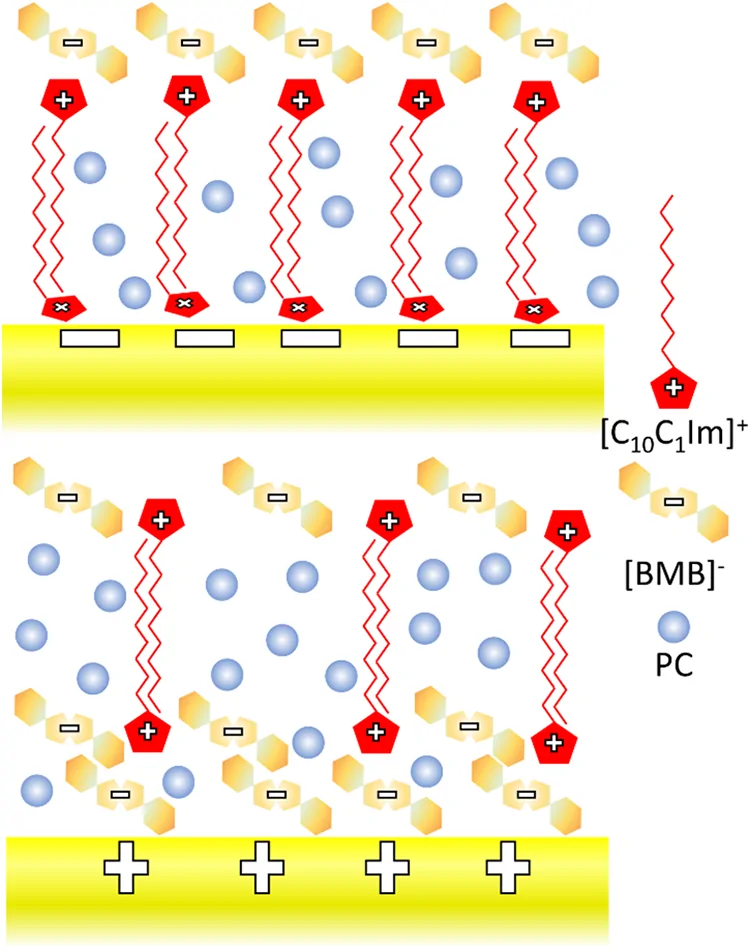
Visual representation of the bilayer structure observed at nonzero potential for [C_10_C_1_Im]­[BMB] at 29% w/w. At negative potential it notes the parallel orientation of the imidazolium ring to the electrode surface and more dense packing of the cation as compared to the positive potential. The size of the [BMB]^−^ anion increases the thickness of the surface layer and the interfacial solvent concentration.

### Effect of Water Bulk Nanostructure and Electro-Driven Interfacial Structuring

Ambient-like levels of D_2_O have previously been added to PC solutions of another nonbilayer-forming IL and represent potential water contaminants at trace levels that might be found in an actual energy storage device.^31,45^ Depending on the bulk IL concentration, the addition of water led to a swelling or thinning of the interface and larger changes in the near interface IL structuring with applied potential. Since bilayer formation is an intrinsically self-assembly process between polar and nonpolar regions, it is instructive to investigate how the presence of water affects this process.

The introduction of trace amounts of water (1% w/w D_2_O) induced very little change in the bulk structuring of the pure ILs (Figure S8), as shown by overlaying the ‘dry’ fits on the hydrated data sets. This observation indicates that the overall nanoscale organization of the IL is largely robust at such levels of hydration. Importantly, the previously discussed enhancement of self-assembly supported by the [BOB]^−^ anion is preserved even in the presence of water, suggesting that its amphiphilic character and ability to promote domain formation are not significantly disrupted by ambient concentrations of water absorption that may be observed in a hygroscopic solvent.

At the surface, however, water has previously been shown to have more significant effects on the interfacial structuring.^31^ Accordingly, a lower amount of water doping was employed; 0.1% w/w D_2_O was added to 20% w/w solutions of [C_10_C_1_Im]­[BOB] and [C_10_C_1_Im]­[BMB] in PC (Figures S9–S11). D_2_O is employed as its high SLD creates contrast against the IL ions, which have lower SLDs (cf. Table 1). This corresponds to 1:10 and 1:8 mol ratios for water:IL pair respectively in the [BOB]^−^ and [BMB]^−^ solutions. In the case of 20% w/w [C_10_C_1_Im]­[BOB] in PC, 0.5% w/w D_2_O was also added (Figures S9–S11). Surface charge and CV plots are presented in the SI, respectively (Figures S12 and S13).

For both the 20% w/w [C_10_C_1_Im]­[BOB] and [C_10_C_1_Im]­[BMB] solutions, the addition of water leads to a more pronounced minimum in SLD at the gold electrode interface at 0 V (cf. Figures S14 and S15). For the 20% w/w [C_10_C_1_Im]­[BOB] solutions, depending on the bulk D_2_O concentration, the thickness of this inner layer varies from ∼13–19 Å (cf. Table S3). In comparison to the characterized structuring of the pure IL on gold,^23^ this thickness range corresponds well to that expected for a cation bilayer (without a counterion anion layer). This layer is also notably thinner that the thickness of the single minimum in the SLD profile of the dry solution (∼30 Å; cf. Figure 4b), which can be assumed to include a (counterion) anion-rich layer that cannot be distinguished from the cation bilayer in the absence of D_2_O due to its more comparable SLD (cf. Table 1).

With increasing D_2_O concentration, this inner layer appears to swell (cf. Table S3), except at the positive potential, consistent with observations from direct force measurements for similar IL bilayers at a negatively charged mica interface.^59^ Due to the presence of D_2_O at the interface, it is likely that a higher dielectric environment ensues, resulting in a greater screening between the cation imidazolium rings and higher packing density. The observed swelling, or thickening, of the cation bilayer thus suggests that this increased packing of the cation rings leads to a reduced degree of interdigitation between the alkyl tails as they become more laterally confined.

Previous atomistic MD simulations for a trihexyltetradecyl phosphonium IL have revealed water to preferentially coordinate with hydrophilic carbonyl groups of the [BOB]^−^ anions to facilitate hydrogen bonds.^60^ Here, however, the imidazolium cation centers can also hydrogen bond with water.^61,62^ Moreover, the thickness of this secondary layer is much larger than expected for the [BOB]^−^ anion, and is instead more comparable to the length of the [BOB]^−^ anion plus two imidazolium rings, orientated perpendicular to the interface. The SLD profile thus suggests that this second layer consists of an intercalated layer of D_2_O-anion complexes between two solvated layers of cation rings.

With applied potential, it is evident that the low SLD inner layer remains at the gold interface. At the positive potential, the SLD of this layer increases in SLD, indicative of a more densely packed layer of the cation’s alkyl chains at the negative potentials (cf. Table 1).

In the case of 20% w/w [C_10_C_1_Im]­[BMB] with 0.1% w/w D_2_O added, similar to that observed for the [C_10_C_1_Im]­[BOB] water solutions, a first (inner) layer of low SLD is present next to the gold interface at all the potentials studied (cf. Table S2). However, the thickness of this layer is typically thinner than with [BOB], particularly toward the negative potentials, suggestive of a denser packing of the cation alkyl chains. Together with the lower SLD of this layer, this suggests that in the case of [BMB]^−^ the alkyl chains of the cations remain interdigitated, while in the [C_10_C_1_Im]­[BOB] solutions the degree of interdigitation decreases in the presence of water.

Previous MD simulations for the same anions, but with a phosphonium-based cation, have suggested that the phenyl rings on the [BMB]^−^ anions can disrupt local hydrogen bonding interactions with water molecules.^60^ Moreover, the water molecules are found to be concentrated around the central polar parts of the anion, while for [BOB]^−^ water molecules are expected to be predominately distributed around the outer carboxyl groups. Therefore, in the case of [BMB]^−^, the presence of water is less likely to lead to a denser packing of the cation rings (i.e., the local dielectric constant remains largely unchanged) and thus reduces the tendency toward less interdigitation observed for the cation alkyl chains with a [BOB]^−^ anion. Both SANS and NR measurements corroborate the same stable self-assembly behavior, irrespective of trace water “contamination”.

## Conclusions

The structuring of two imidazolium orthoborate ILs bearing a long alkyl chain (*n* = 10) has been studied in the bulk and at a gold electrode as a function of their bulk concentration in a polar solvent, PC. The observed interfacial and bulk assemblies were resilient to the addition of trace water. For both ILs, a distinct interfacial region of lower SLD than that of the bulk IL solution was observed, which varied in its extent and composition depending on the applied potential to the gold electrode. The long (nonpolar) alkyl chain of the cation gives rise to self-assembly behavior, where the cations assemble into interdigitated bilayers at charged interfaces.^23^ Here, for all conditions, the thickness of the interfacial region is larger than expected for one interdigitated bilayer. Instead, excluding large surface potentials, the results suggest that two interdigitated bilayers reside at the gold interface with the anions largely intercalated between the (polar) cation imidazolium rings. However, the degree of partitioning of the interface into these nonpolar and polar regions is more pronounced for the larger anion [BMB]^−^. An effect of size exclusion as well as the presence of additional π-π interactions between the [BMB]^−^ phenyl rings and cation imidazolium rings playing a contributing role.

SANS measurements support the findings of NR, mirroring the observed interfacial trends with bulk information. Namely, [C_10_C_1_Im]­[BOB] was a universally superior self-assembling IL, with higher aggregation numbers and a concentration-independent negative amphiphile parameter. Conversely, the large size and hydrophobic phenyl appendages of [BMB]^−^ disrupt the stability of the self-assembly structures. This behavior is facilitated by additional π-π interactions in the same way as upon the electrode.

At low concentrations, the structuring of the two ILs is similar, with the observed changes in SLD and interfacial thickness following a pseudolinear trend with applied potential, inferring an ion-exchange charge compensation mechanism at the interface. Such behavior has been associated with a high-volume fraction of the dielectric solvent PC within the literature. The PC screens the ion pairs, enhancing ion mobility. For higher bulk concentrations, the different molecular structures of the two anions appear to play a larger role in determining the interfacial structuring of the ILs: For the smaller anion [BOB]^−^, the results at an intermediate concentration point toward a multilayer structuring, that is not observed for [BMB]^−^.

Together, these comprehensive experimental results demonstrate that cationic IL bilayers can perform as robust electroactive layers, whose interfacial structuring and composition can be intricately tuned through electric fields. Moreover, such interfacial bilayers are found to retain their structuring in the presence of ambient levels of water. However, the choice of anion and bulk IL concentration are shown to play an important role in the charge compensating mechanisms of the IL layers.

The chemical control of the bulk and electroactive self-assembly of ILs in PC, and the interaction with water “contamination” has profound implications in the design of IL based supercapacitors, as well as electroactive lubricants in polar media.

## Supplementary Material



## Notes

Experimental details describing the NR methodology; NR analysis; calculated surface charge; cyclic voltammograms and SANS fits of experimental data (PDF)

## ●

Neutron Scattering Division, Oak Ridge National Laboratory, Oak Ridge, Tennessee, USA
