# Supplementary material for: Self-Assembly Versus Surface Charge: Interfacial Response of Ionic Liquids
Source: Langmuir. 2026 Sep 16;42(38):28123–34. doi: 10.1021/acs.langmuir.6c03265 (PMC13629669; doi:10.1021/acs.langmuir.6c03265)
Supplement: Supplementary file 1 [file la6c03265_si_001.pdf]

Supplementary Information for "Self-assembly versus surface charge: Interfacial response of ionic liquids"

Georgia A. Pilkington[a], Oliver S. Hammond[a,b,c,d,e], Erik Bergendal[a], Anna Oleshkevych[a], Stuart J. Brown[a,f], Sichao Li[a], Andrew Nelson[g], Liliana de Campo[g], Rebecca Welbourn[h,i], Akepati Bhaskar Reddy[j], Manishkumar R. Shimpi[c], Sergei Glavatskih,[j,k,l] Anja-Verena Mudring[c,d] and Mark W. Rutland[a,e,f,l,m,*]

[a]Division of Surface and Corrosion Science, School of Engineering Sciences in Chemistry, Biotechnology and Health, KTH Royal Institute of Technology, SE-100 44 Stockholm, Sweden. [b]European Spallation Source, Data Management & Scientific Computing, Asmussens Allé 305, Kongens Lyngby, 2800, Hovedstaden, Denmark [c]Department of Materials and Environmental Chemistry, Stockholm University, Stockholm SE-114 18, Sweden. [d]Department of Biological and Chemical Engineering, Aarhus University, Aarhus N 8200, Denmark. [e]School of Chemistry, University of New South Wales, Sydney, NSW 2052, Australia [f]Wallenberg Initiative Materials Science for Sustainability, Division of Surface and Corrosion Science, KTH Royal Institute of Technology, SE-100 44, Stockholm, Sweden. [g]Australian Nuclear Science and Technology Organisation (ANSTO), Lucas Heights, Australia, NSW 2234. [h]ISIS Pulsed Neutron and Muon Facility, Rutherford Appleton Laboratory, Didcot, Oxfordshire OX11 0QX, UK [i]current address: Neutron Scattering Division, Oak Ridge National Laboratory, Oak Ridge, Tennessee, USA [j]System and Component Design, Department of Machine Design, KTH Royal Institute of Technology, SE-100 44 Stockholm, Sweden. [k]Department of Electromechanical, Systems and Metal Engineering, Ghent University, B-9052 Ghent, Belgium. [l]Surfaces, Processes and Formulation, RISE Research Institutes of Sweden, SE-100 44 Stockholm, Sweden. [m]Department of Physics, Umeå University, 907 36 Umeå, Sweden. [n]Laboratoire de Tribologie et Dynamique des Systèmes, École Centrale de Lyon, Écully, Lyon, 69134, Auvergne-Rhône-Alpes, France.

**Corresponding author: mark@kth.se*

Table of Contents

Neutron Reflectivity Methodology 2
Gold in Air Reflectometry 3
Surface Charge Density 4
Cyclic Voltammetry 5
NR Asymmetry Plots 6
NR SLD Profiles 8
NR Fitting Parameters 10
SANS Fitting 11
D_2O Additive NR 12
D_2O Additive Asymmetry Plots 13
D_2O Additive Surface Charge 15
D_2O Additive Cyclic Voltammetry 15
D_2O Additive SLD Plots 16
D_2O Additive NR Fitting Parameters 18

Neutron Reflectivity Methodology

NR Solid-Liquid Electrochemical Cell Design

Before assembling the cell, the conductive glass (coated with fluorine-doped tin oxide, FTO; NSG TEC A7, Pilkington, UK) used as counter electrode, as well as all the other cell components, were sonicated in 2% Hellmanex™ for 30 minutes, before being rinsed thoroughly with Milli-Q water and absolute ethanol, and dried with filtered N_2. For the measurements presented here, a PTFE gasket of 0.5 mm thickness was used to separate the working (gold films) and counter electrodes. We choose to use a two-electrode system to avoid potential impurities, instability and internal resistance issues that may arise from use of Pt or Ag wires, which are commonly used as quasi-reference electrodes in non-aqueous systems.[1] In order to minimise ambient water contamination, the cell was assembled and subsequently filled inside a polyethylene glove bag under a dry, nitrogen atmosphere (R.H. < 10%). The IL solutions were injected into the bottom port of the cell through PTFE tubing using a glass Luer syringe. Once mounted on the beamline, electrical potentials were applied across the NR cell using a potentiostat (either a Metrohm Autolab 302 or PGSTAT204 at ANSTO or ISIS respectively).

NR Experimental Parameters

For NR experiments conducted on the Platypus beamline (OPAL, Australia) two incident angles (0.65° and 3°) were used to cover the Q-range from critical edge to background (0.008 – 0.22 $Å^{-1}$). A fixed dQ/Q resolution was used for all measurements (approx. 5%). In the case of the INTER experiments at ISIS (Rutherford Appleton Laboratory, England), the reflectivity measurements were also measured at two incident angles (0.5° and 1.8°), providing a Q-range of 0.0065-0.22 $Å^{-1}$, and the resolution (dQ/Q) was selected to be around 4%. All the scattering data were reduced using the standard procedures. The datasets had a background subtraction, where applicable, detector efficiency calibration, followed by normalisation to reflectivity by the direct beam (a transmission measurement through the silicon blocks). Datasets from the two angles were stitched together to form one profile.

Micro-slab Fitting Technique

The micro-slab fitting method splits the interfacial region into a series of 1 Å thick slabs of 0 Å roughness. The SLD of each micro-slab is determined using a normal distribution of the volume fraction of Au and the first IL layer to describe a smooth transition between these regions. The only fitted parameter is the thickness of this interfacial region (t_i), which replaces the gold interfacial roughness parameter. The cumulative distribution function in SciPy[2] was used to calculate the normal distribution, with the scale set to $3t_i/20$ to ensure the volume fraction approached 0 and 1 at the edges of t_i.

Gold in Air Reflectometry

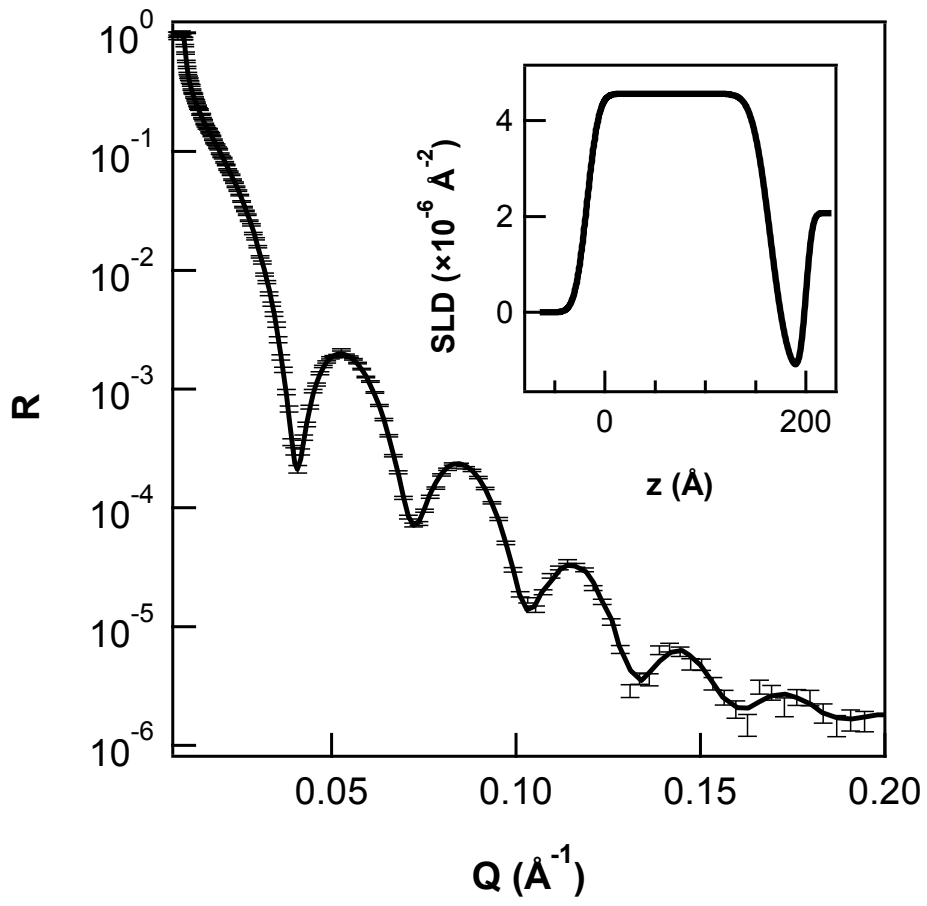

Figure S1. Representative reflectivity of a gold electrode surface in air before they were used for NR measurements on the IL solutions. This specific gold electrode surface was used for the solution 5% w/w [$C_{10}C_1$Im][BOB] in PC, the results from which are presented in Figure 2a.

Table S1. Fitted parameters obtained from a best-fit to the reflectivity of the representative gold substrate measured in air and presented in Fig. S1. *Calculated theoretical values.

Layer	Thickness (Å)	SLD (×10^{-6} Å^{-2})	Roughness (Å)
Au	183.0 ± 0.5	4.56*	9.4 ± 0.5
SiO_2/Ti	35.3 ± 0.5	-1.37 ± 0.01	14.1 ± 0.4
Si	∞	2.07*	5.1 ± 0.6

Surface Charge Density

(a)

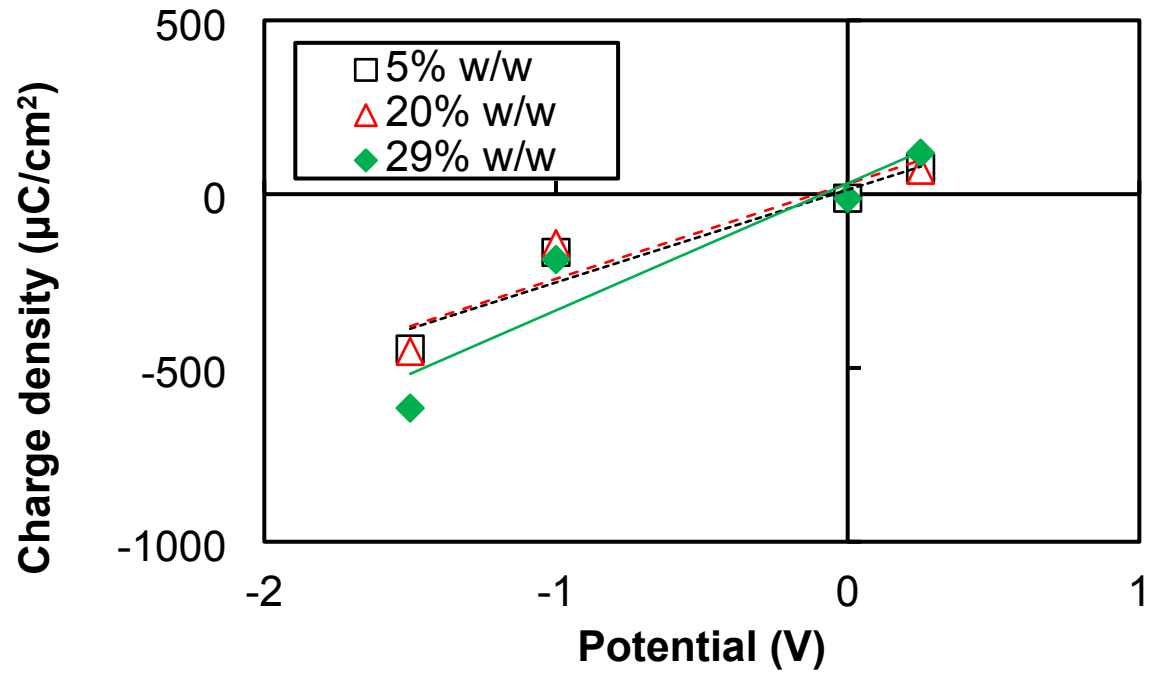

(b)

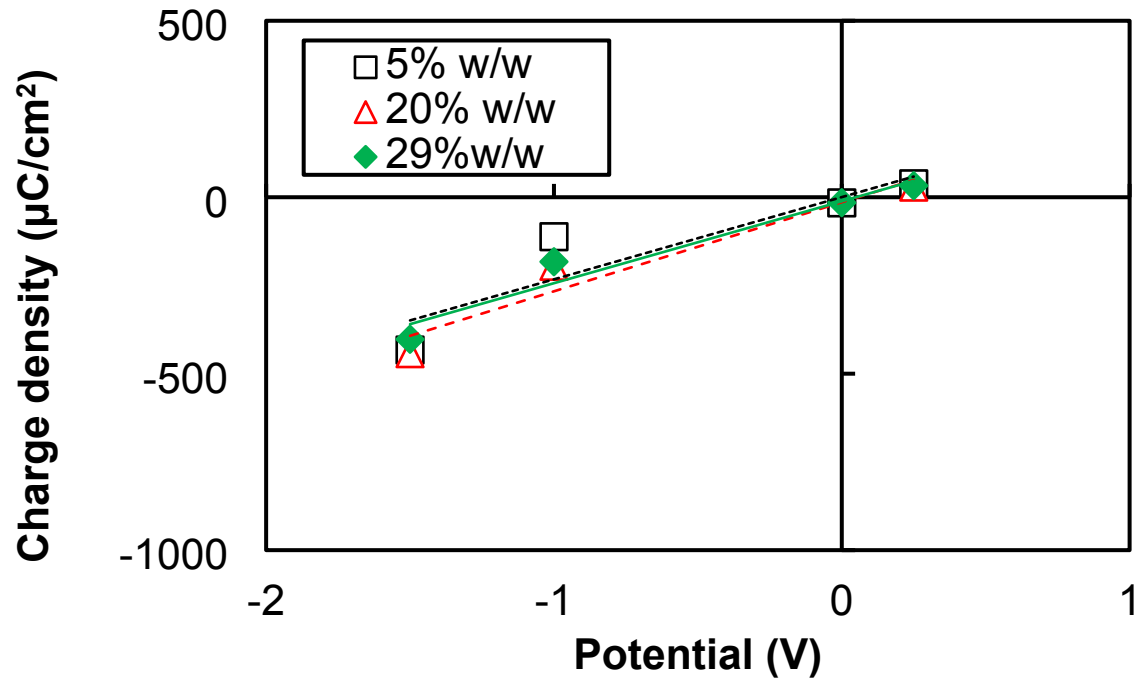

Figure S2. Surface charge densities for different concentrations of **(a)** [$C_{10}C_1$Im][BOB] and **(b)** [$C_{10}C_1$Im][BMB] in PC. All surface charge densities are estimated from in-situ chrono-amperometry measurements during the 30 minute stabilisation period allowed after application of different applied potentials before NR measurements. The straight lines are linear fits as guides to the eye and to provide a linear comparison.

Cyclic Voltammetry

(a)

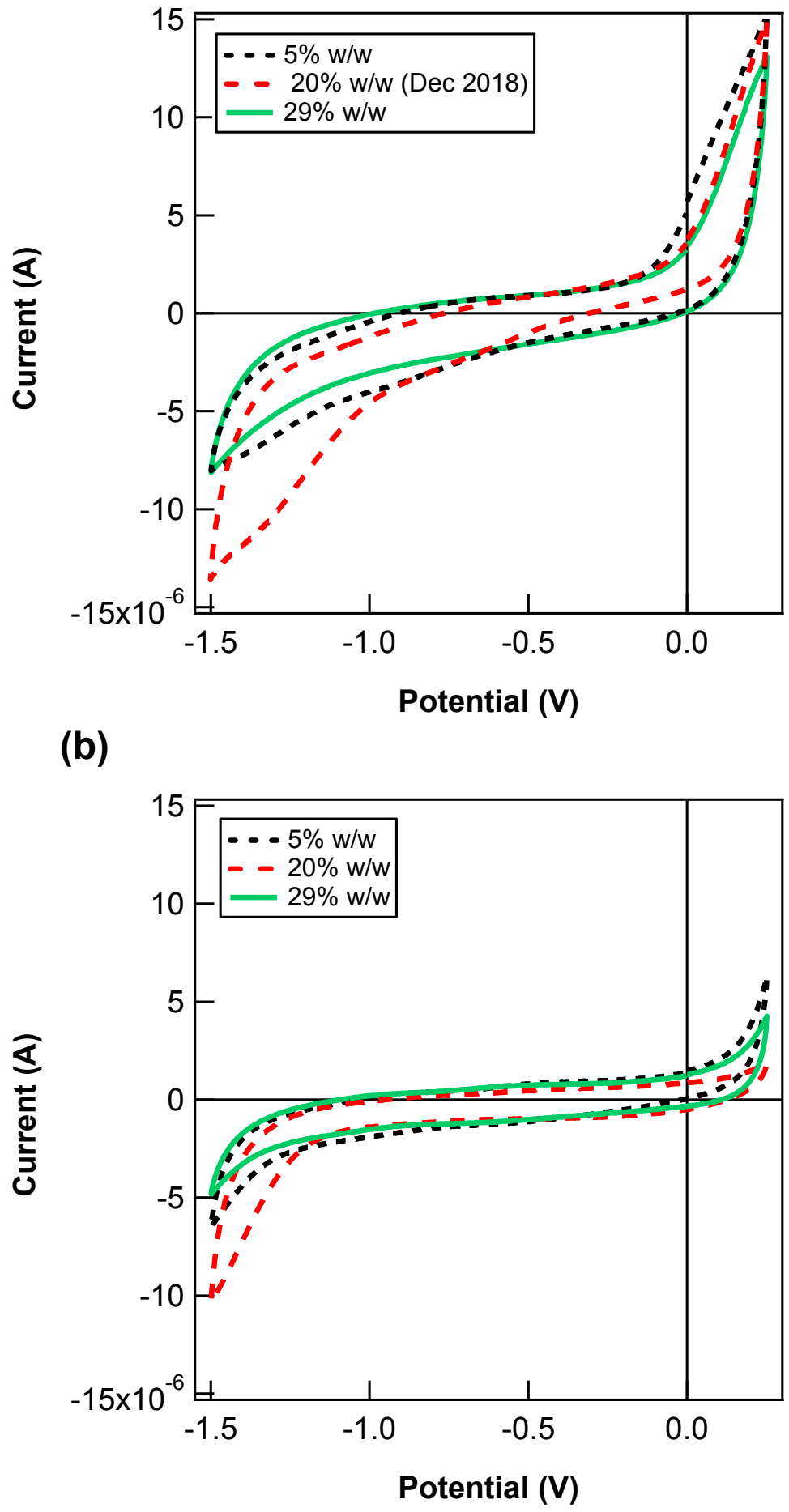

Figure S3. Cyclic voltammograms (CVs) for different concentrations of **(a)** $[C_{10}C_1Im][BOB]$ and **(b)** $[C_{10}C_1Im][BMB]$ in PC. All the CVs were performed in the electrochemical NR cell at a potential sweep rate of 10 mV/s after NR measurements. The potential ranges correspond to those investigated by NR.

NR Asymmetry Plots

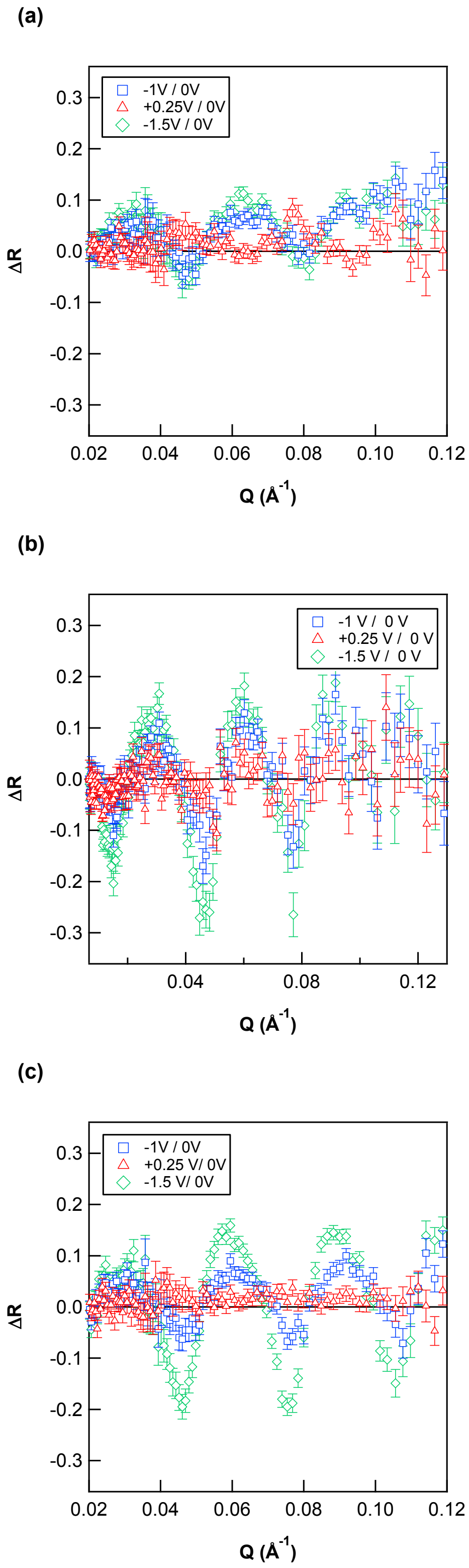

Figure S4. Asymmetry plots for **(a)** 5, **(b)** 20 and **(c)** 29% w/w solutions of [$C_{10}C_1$Im][BOB] in PC, where $\Delta R = [R^V(Q_z)-R^0(Q_z)]/[R^V(Q_z)+R^0(Q_z)]$, obtained from the experimental data, highlighting the changes in the non-zero potential reflectivities (R^v) with respect to 0 V (R^0). The size of the error bars vary between (a)-(c) as the data for (a) and (c) were collected on the reflectometer Platypus at ANSTO, Australia, whilst (b) was collected on the reflectometer INTER at ISIS, UK.

(a)

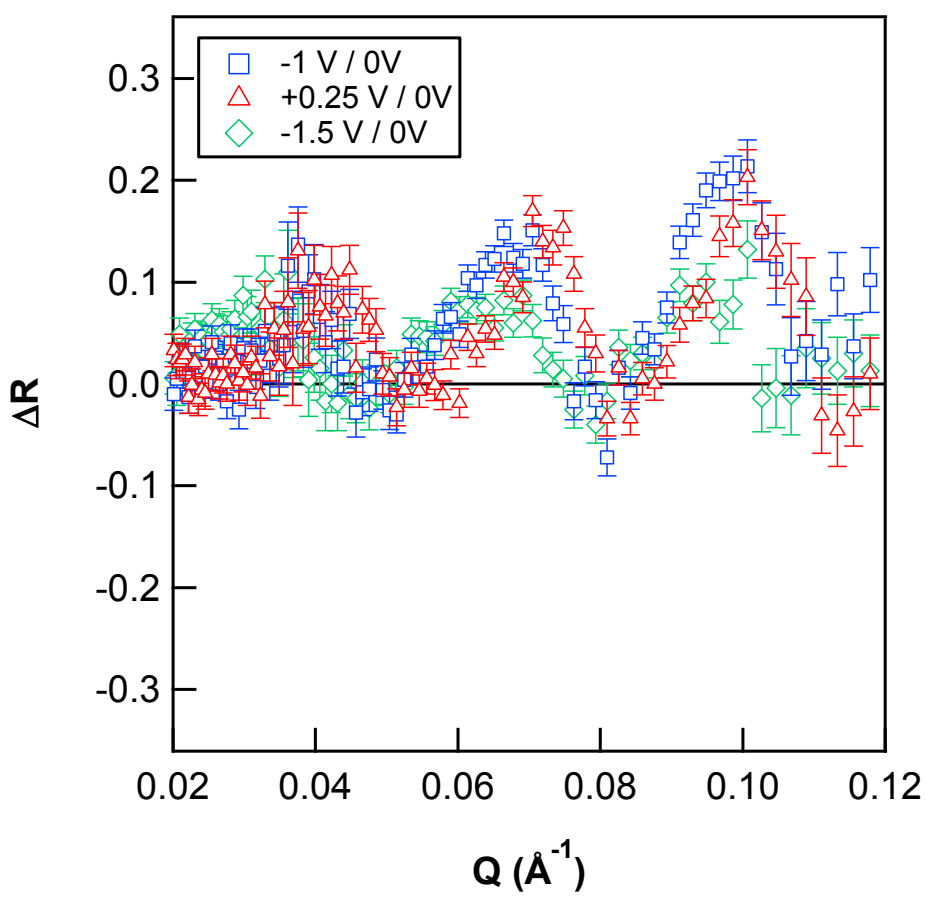

(b)

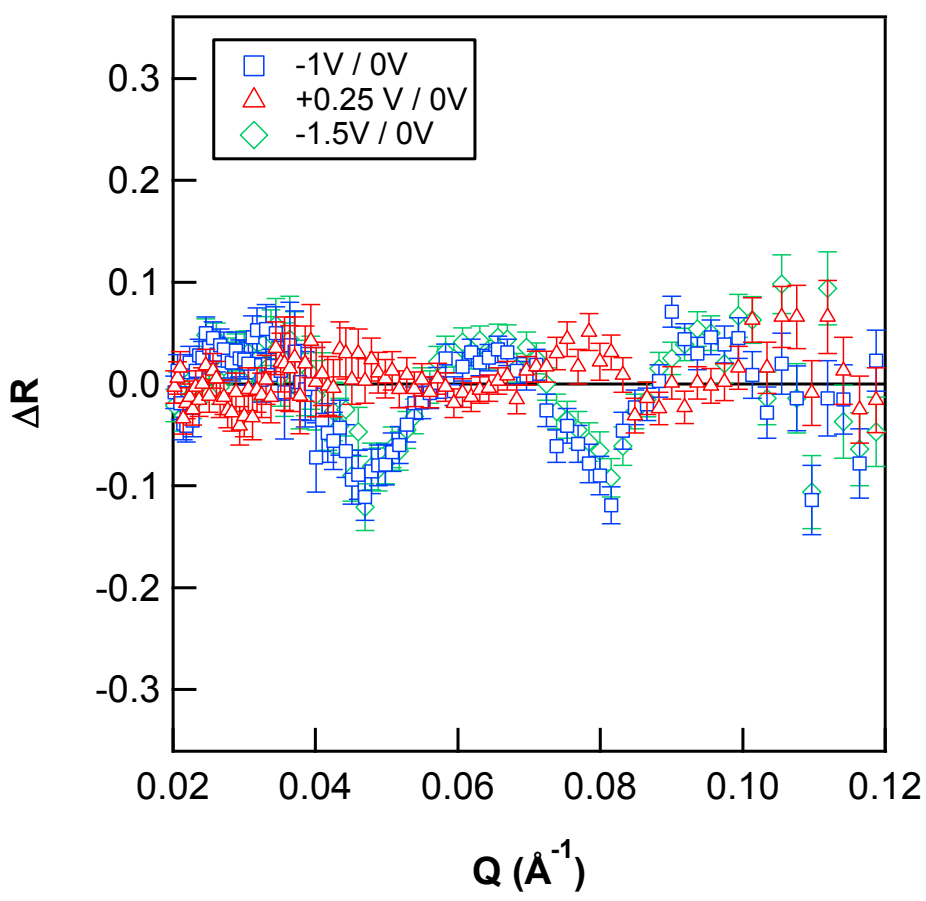

Figure S5. Asymmetry plots for **(a)** 5 and **(b)** 29% w/w solutions of [$C_{10}C_1$Im][BMB] in PC, where ΔR = [$R^V(Q_z)$-$R^0(Q_z)$]/[$R^V(Q_z)$+$R^0(Q_z)$], obtained from the experimental data, highlighting the changes in the non-zero potential reflectivities (R^v) with respect to 0 V (R^0). 0 V was not measured for 20% w/w due to time limitations, so it is not possible to present the asymmetry plots for this concentration.

NR SLD Profiles

(a)

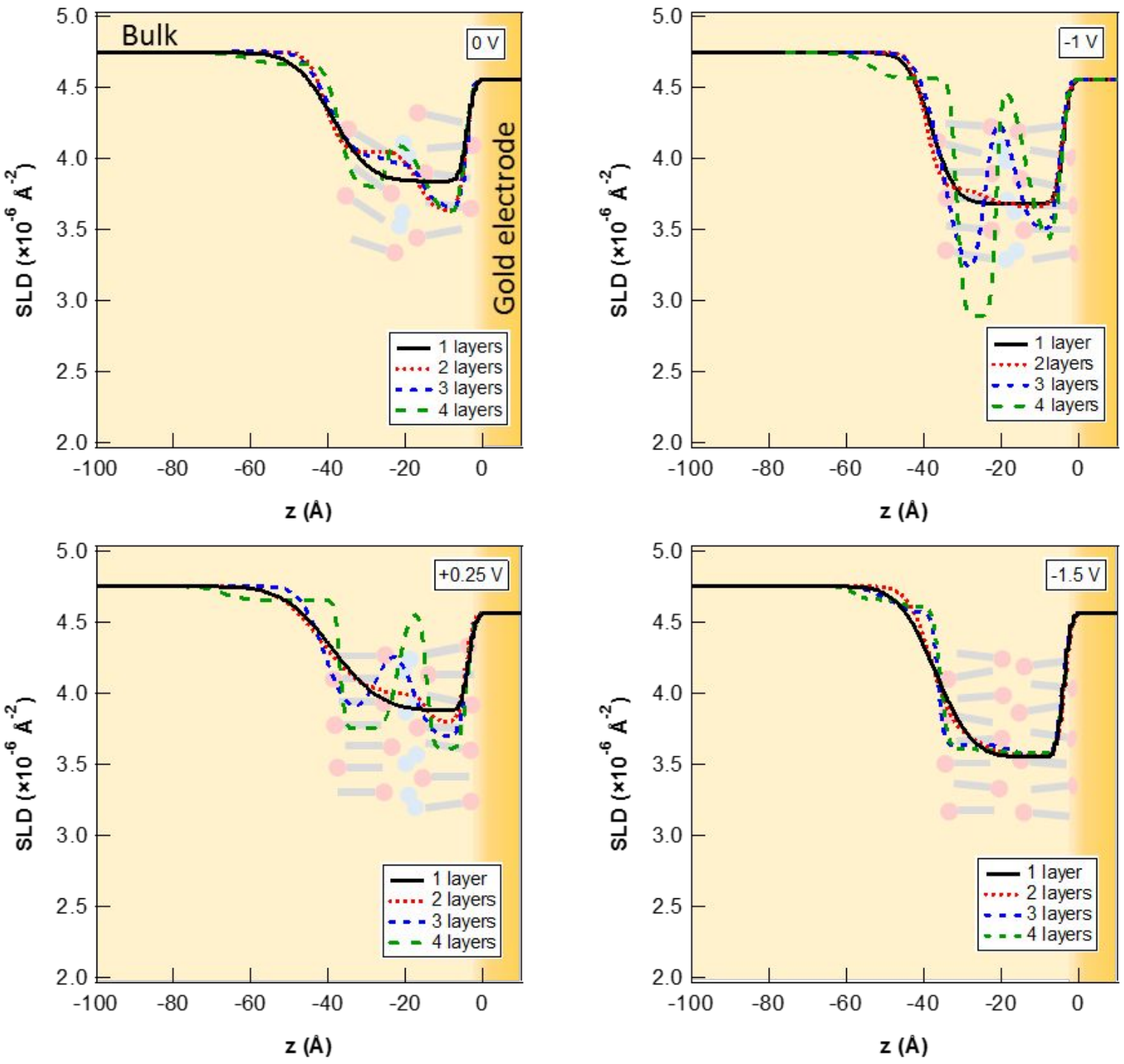

(b)

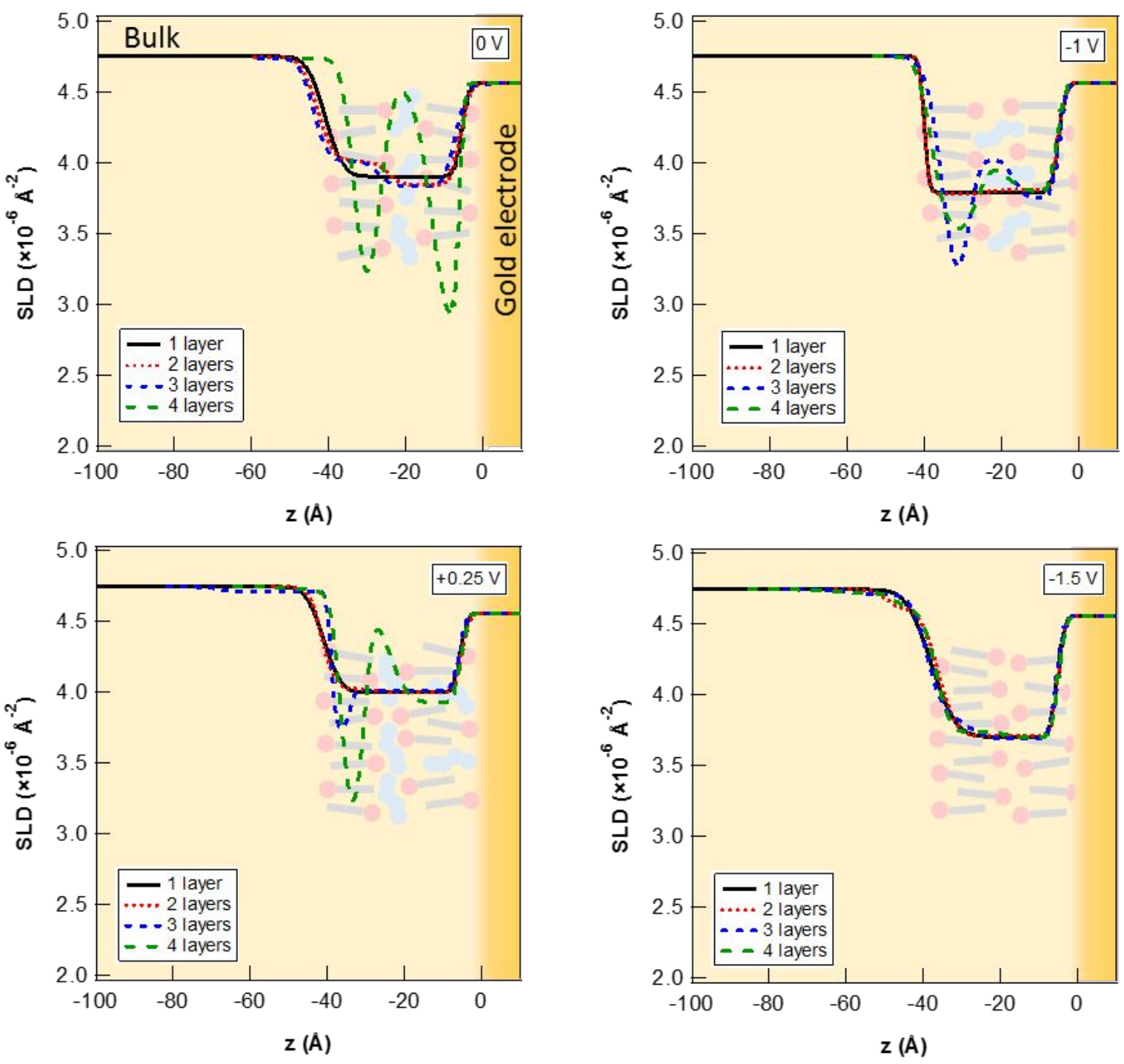

Figure S6. SLD profiles for 5% w/w **(a)** [$C_{10}C_1$Im][BOB] and **(b)** [$C_{10}C_1$Im][BMB] in PC at different applied potentials obtained from the best-fits using different numbers of layers, where the gold-IL interface is located at z = 0. In all cases a 1-layer model was deemed to provide the best-fit to the data as it had the lowest FOM or the addition of subsequent layers did not improve the FOM significantly. The SLD profiles with multiple layers do however suggest variations in the SLD that are not captured in the 1-layer model and are suggestive of well-defined regions of lower and higher SLD corresponding to high volume fractions of the cation alkyl chains, and cation imidazolium ring and anions, respectively. Schematic structuring of the IL ions at the gold interface are shown based on these variations.

(a)

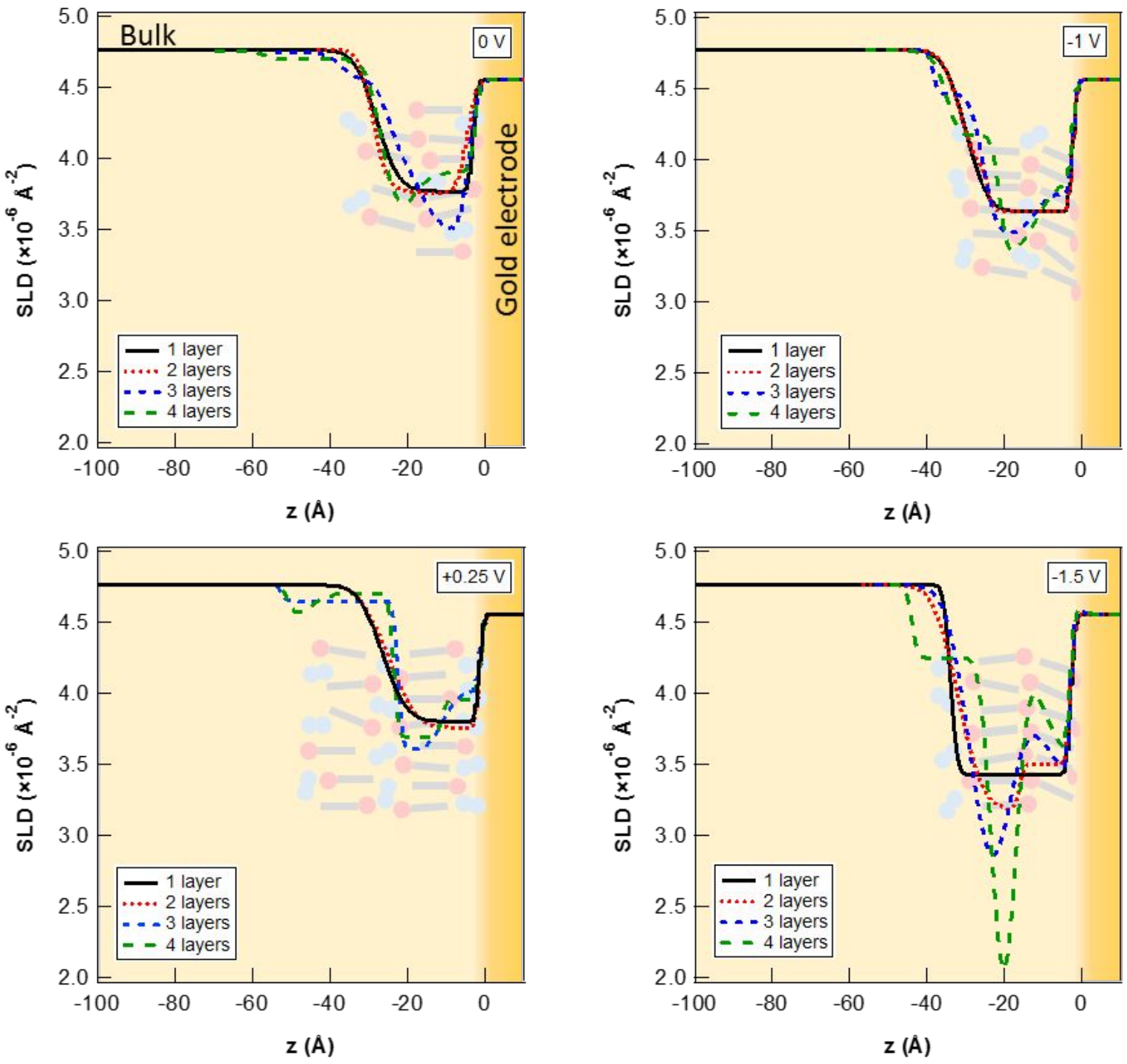

(b)

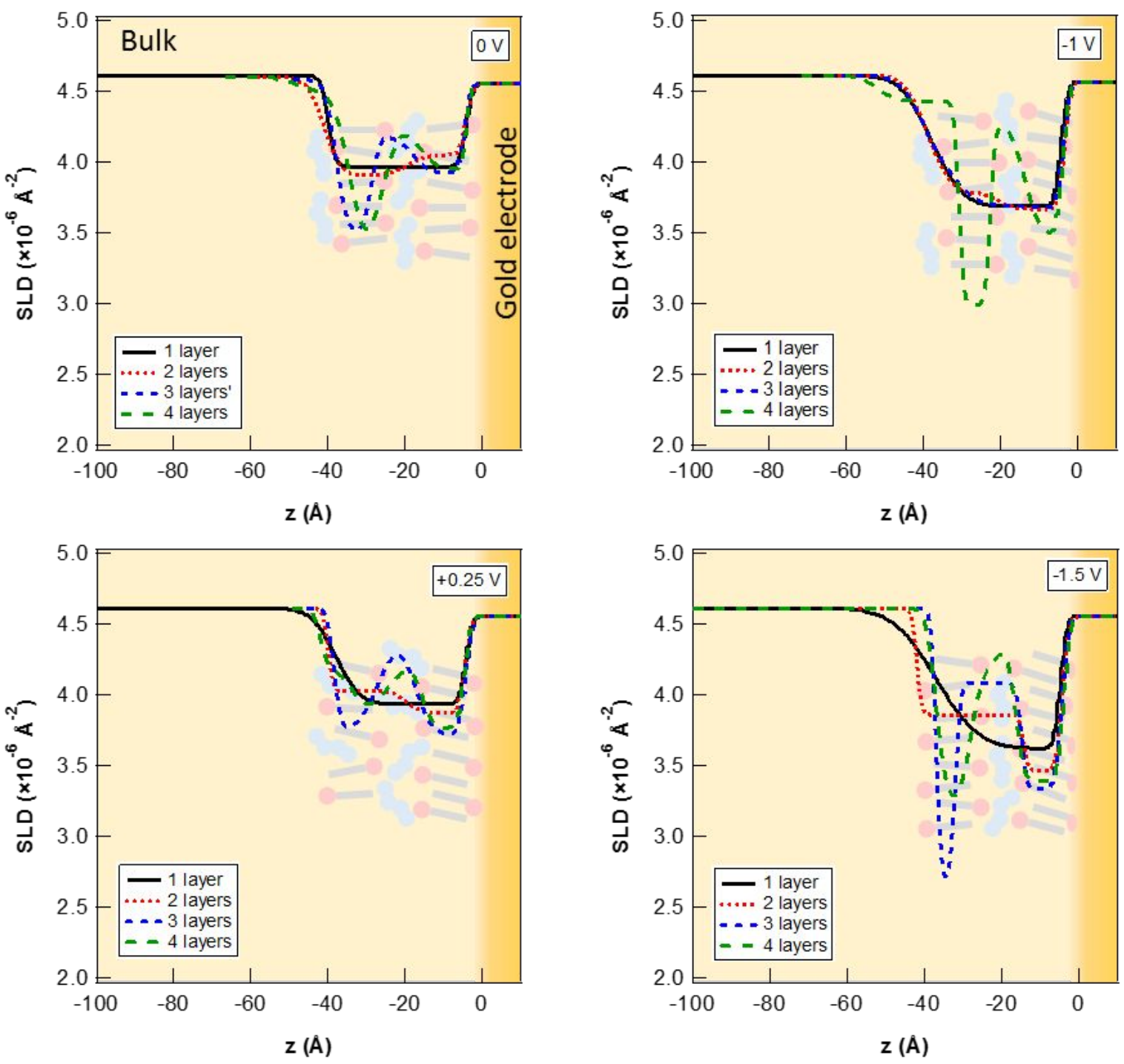

Figure S7. SLD profiles for 29% w/w **(a)** [$C_{10}C_1$Im][BOB] and **(b)** [$C_{10}C_1$Im][BMB] in PC at different applied potentials obtained from the best-fits using different numbers of layers, where the gold-IL interface is located at z = 0. In all cases a 1-layer model was deemed to provide the best-fit to the data as it had the lowest FOM or the addition of subsequent layers did not improve the FOM significantly. The SLD profiles with multiple layers do however suggest variations in the SLD that are not captured in the 1-layer model and are suggestive of well-defined regions of lower and higher SLD corresponding to high volume fractions of the cation alkyl chains, and cation imidazolium ring and anions, respectively. Schematic structuring of the IL ions at the gold interface are shown based on these variations.

NR Fitting Parameters

Table S2. Best-fit parameters obtained from SLD models to NR reflectivities for the ILs [$C_{10}C_1$Im][BOB] and [$C_{10}C_1$Im][BOB] in PC solutions at different bulk IL concentrations and applied potentials at a gold electrode interface. The potentials are presented in chronological ordering of the NR experiment.

IL	Concentration (wt%)	Bulk SLD (10^{-6} Å^{-2})	Voltage (V)	Layer Thickness (Å)	SLD (10^{-6} Å^{-2})	Roughness (Å)
[$C_{10}C_1$Im][BOB]	5	4.75	0	29.3 ± 1.2	3.84 ± 0.05	7.8 ±2.2
			-1	28.7 ± 1.0	3.69 ± 0.05	5.3 ± 2.4
			0.25	29.5 ± 1.6	3.88 ± 0.03	10.0 ± 1.4
			-1.5	27.6 ± 1.1	3.55 ± 0.04	7.5 ± 0.9
[$C_{10}C_1$Im][BOB]	20	4.63	0	23.9 ± 3.2	3.90 ± 0.11	11.1 ± 2.4
			-1	30.7 ± 2.0	3.76 ± 0.06	8.3 ± 1.6
			0.25	25.9 ± 2.5	3.85 ± 0.07	12.0 ± 2.7
			-1.5	33.9 ± 1.3	3.55 ± 0.05	9.9 ± 3.0
[$C_{10}C_1$Im][BOB]	29	4.77	0	21.3 ± 2.1	3.77 ± 0.07	4.5 ± 2.0
			-1	24.2 ± 1.8	3.63 ± 0.11	4.5 ± 1.6
			0.25	21.4 ± 1.8	3.80 ± 0.08	4.9 ± 2.0
			-1.5	27.7 ± 1.5	3.43 ± 0.08	1.4 ± 1.5
[$C_{10}C_1$Im][BMB]	5	4.74	0	30.5 ± 1.9	3.90 ± 0.04	5.3 ± 1.4
			-1	29.6 ± 1.4	3.80 ± 0.05	1.0 ± 2.0
			0.25	30.6 ± 2.0	4.00 ± 0.05	2.9 ± 1.0
			-1.5	27.3 ± 1.0	3.70 ± 0.04	5.8 ± 1.4
[$C_{10}C_1$Im][BMB]	20	4.51	-1	33.0 ± 2.2	3.76 ± 0.03	6.5 ± 1.7
			0.25	33.9 ± 2.8	3.96 ± 0.06	7.6 ± 4.2
			-1.5	29.7 ± 1.8	3.61 ± 0.06	9.8 ± 2.0
[$C_{10}C_1$Im][BMB]	29	4.61	0	31.1 ± 1.2	3.97 ± 0.05	1.5 ± 1.4
			-1	28.6 ± 1.0	3.69 ± 0.05	5.0 ± 2.7
			0.25	28.2 ± 2.4	3.92 ± 0.06	6.0 ± 1.3
			-1.5	27.5 ± 1.7	3.62 ± 0.07	8.8 ± 1.5

SANS Fitting

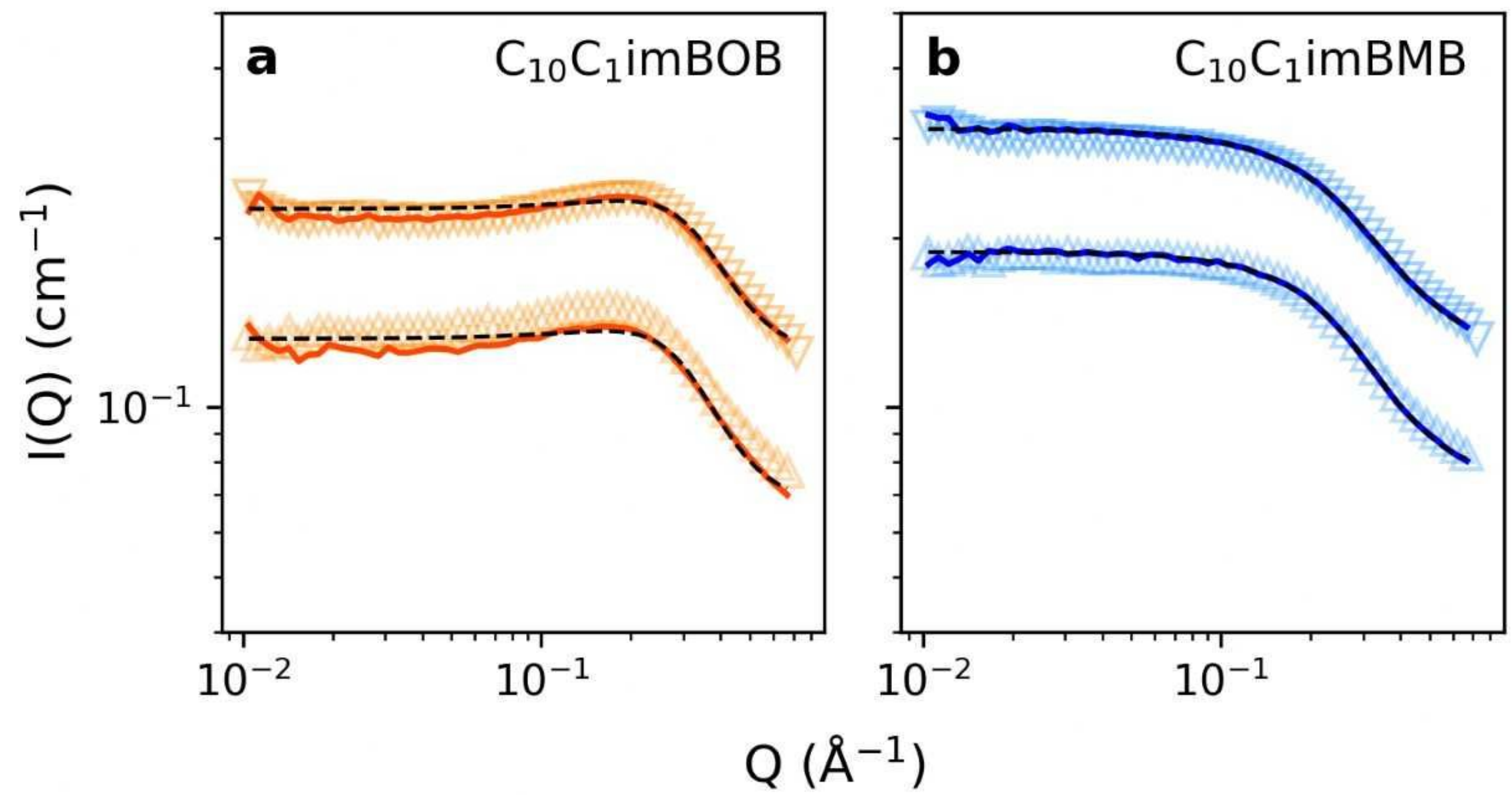

Figure S8. SANS curves showing (**a**) [$C_{10}C_1$Im][BOB] and (**b**) [$C_{10}C_1$Im][BMB] in solution with D-PC at 10% w/w (bottom sets with lower I(Q); upwards oriented triangles) and 20% w/w (top sets with greater I(Q); downwards oriented triangles). In each subplot, the coloured solid lines pertain to the 'pure' data without water, the markers are the 1% w/w D2O samples, and the black dashed lines are the Teubner-Strey fits to the 'pure' dataset.

D_2O Additive NR

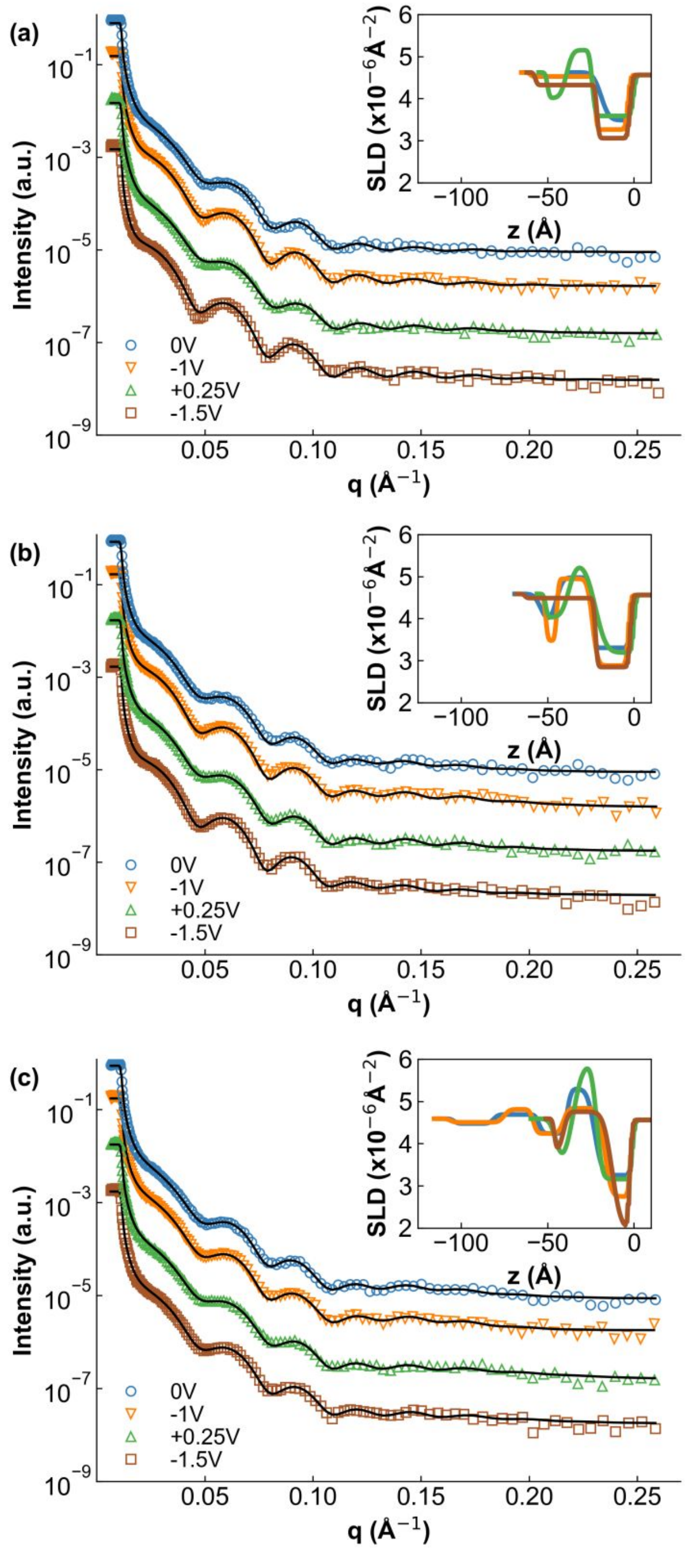

Figure S9. Reflectivity measured for a solution of 20% w/w [$C_{10}C_1$Im][BOB] with **(a)** 0.1 and **(b)** 0.5% w/w added D_2O at different applied potentials at a gold electrode interface. **(c)** Reflectivity measured for a solution of 20% w/w [$C_{10}C_1$Im][BMB] with 0.1 w/w D_2O at different applied potentials at a gold electrode interface. The symbols represent the experimental data, whilst the solid lines represent best-fits to the data. The insets show SLD profiles for different applied potentials obtained from the best-fits presented, where the gold-IL interface is located at z = 0. The potentials are presented in chronological ordering of the NR experiment.

D_2O Additive Asymmetry Plots

(a)

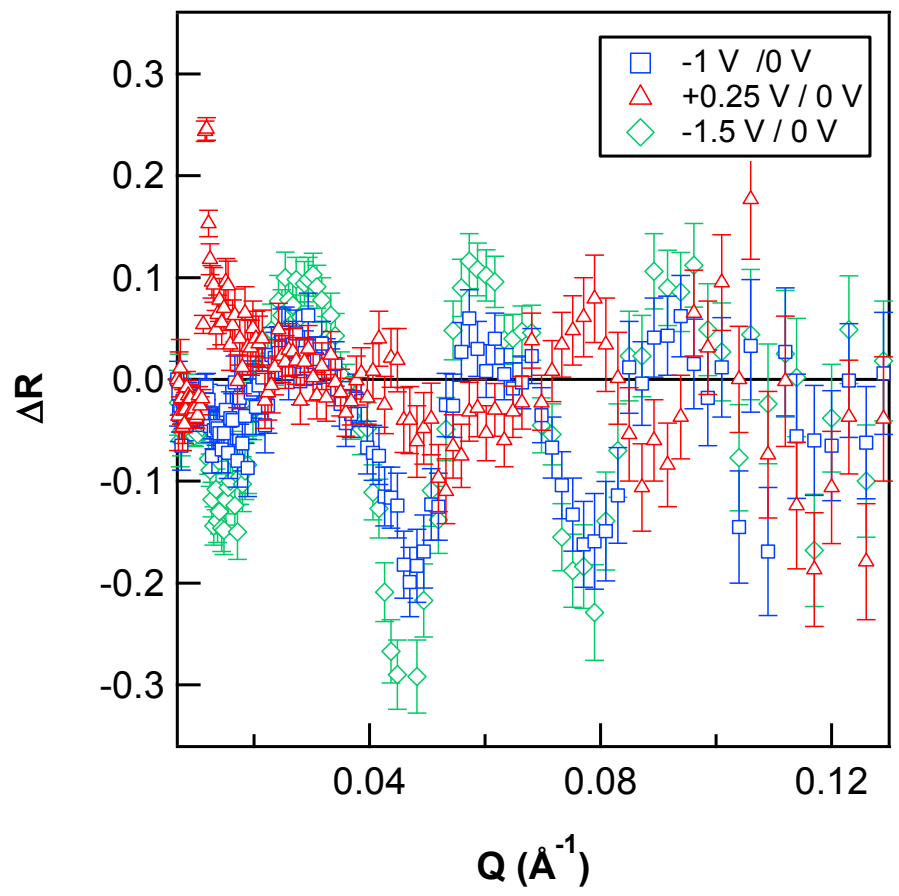

(b)

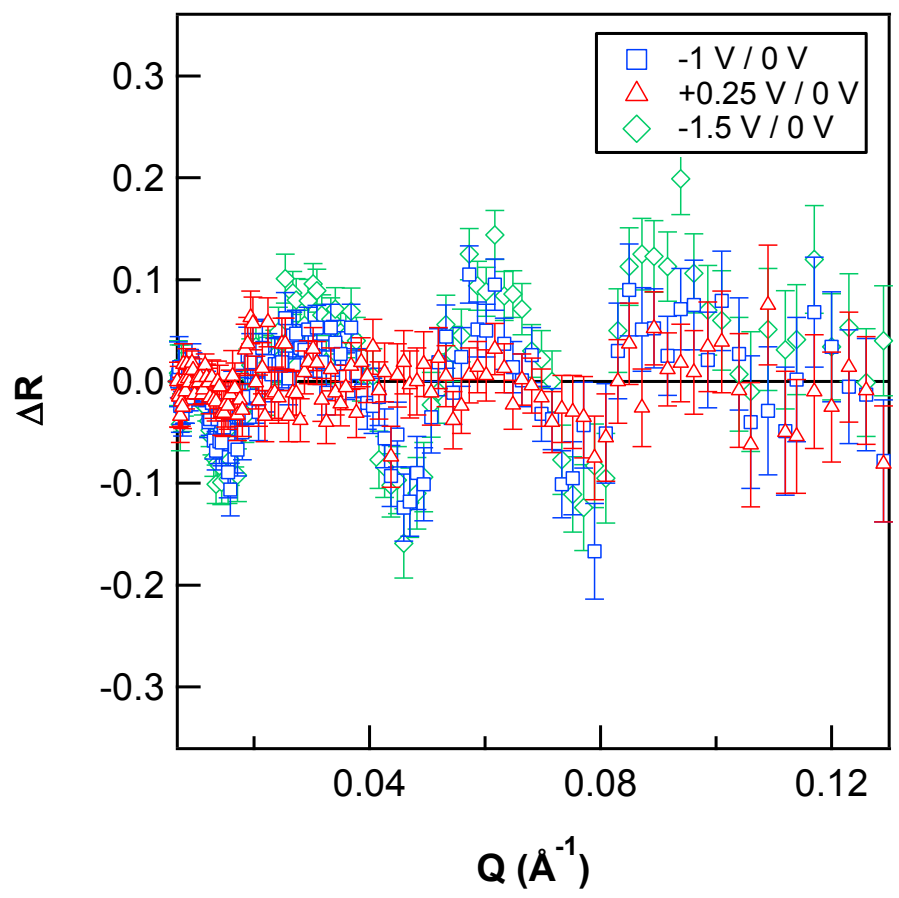

Figure S10. Asymmetry plots for 20% w/w solutions of [$C_{10}C_1$Im][BOB] in PC with **(a)** 0.1 and **(b)** 0.5% w/w D_2O added, where $\Delta R = [R^V(Q_z)-R^0(Q_z)]/[R^V(Q_z)+R^0(Q_z)]$, obtained from the experimental data, highlighting the changes in the non-zero potential reflectivities (R^v) with respect to 0 V (R^0).

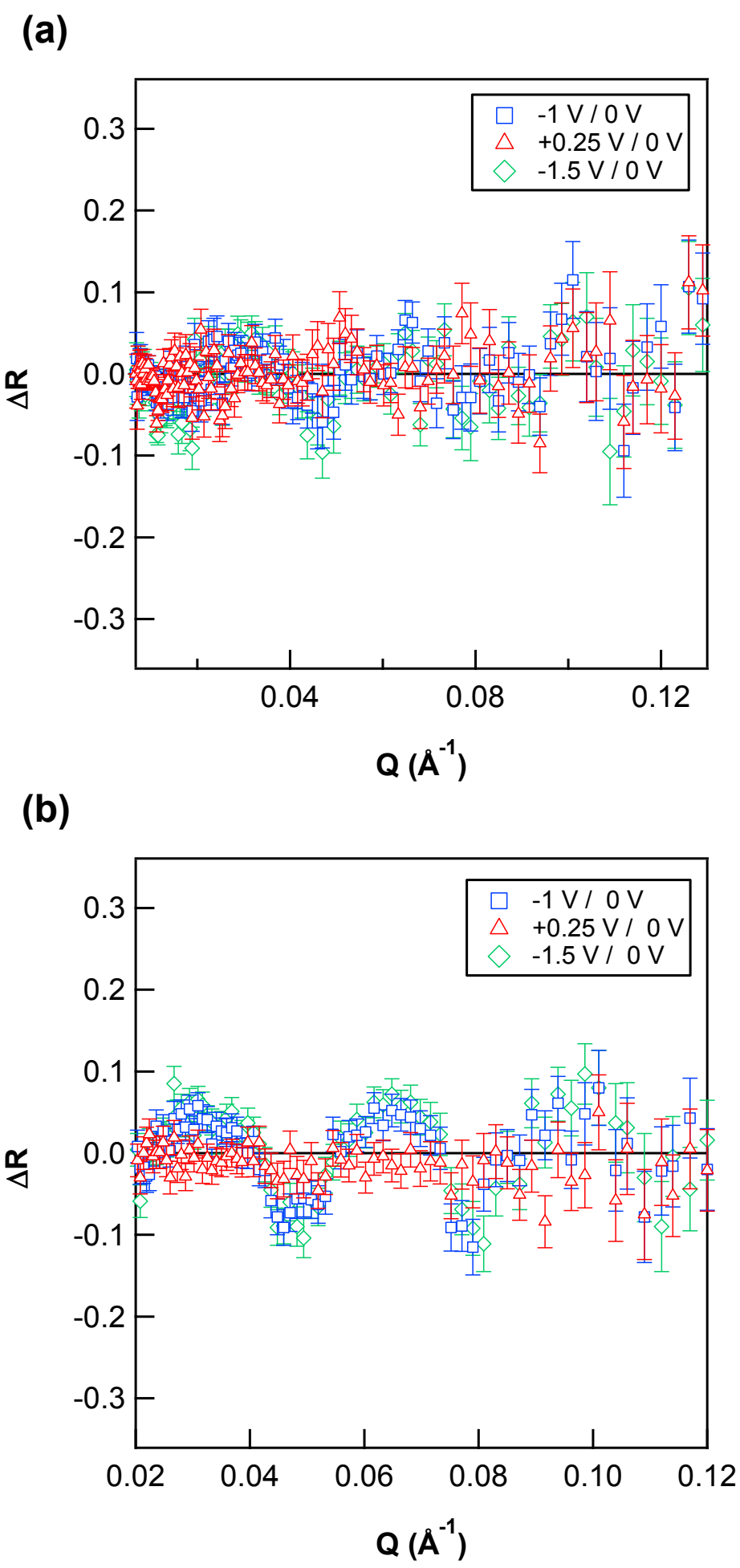

Figure S11. Asymmetry plots for 20% w/w solutions of [$C_{10}C_1$Im][BMB] in PC with **(a)** 0.1 and **(b)** 0.5% w/w D_2O added, where $\Delta R = [R^V(Q_z)-R^0(Q_z)]/[R^V(Q_z)+R^0(Q_z)]$, obtained from the experimental data, highlighting the changes in the non-zero potential reflectivities (R^v) with respect to 0 V (R^0).

D_2O Additive Surface Charge

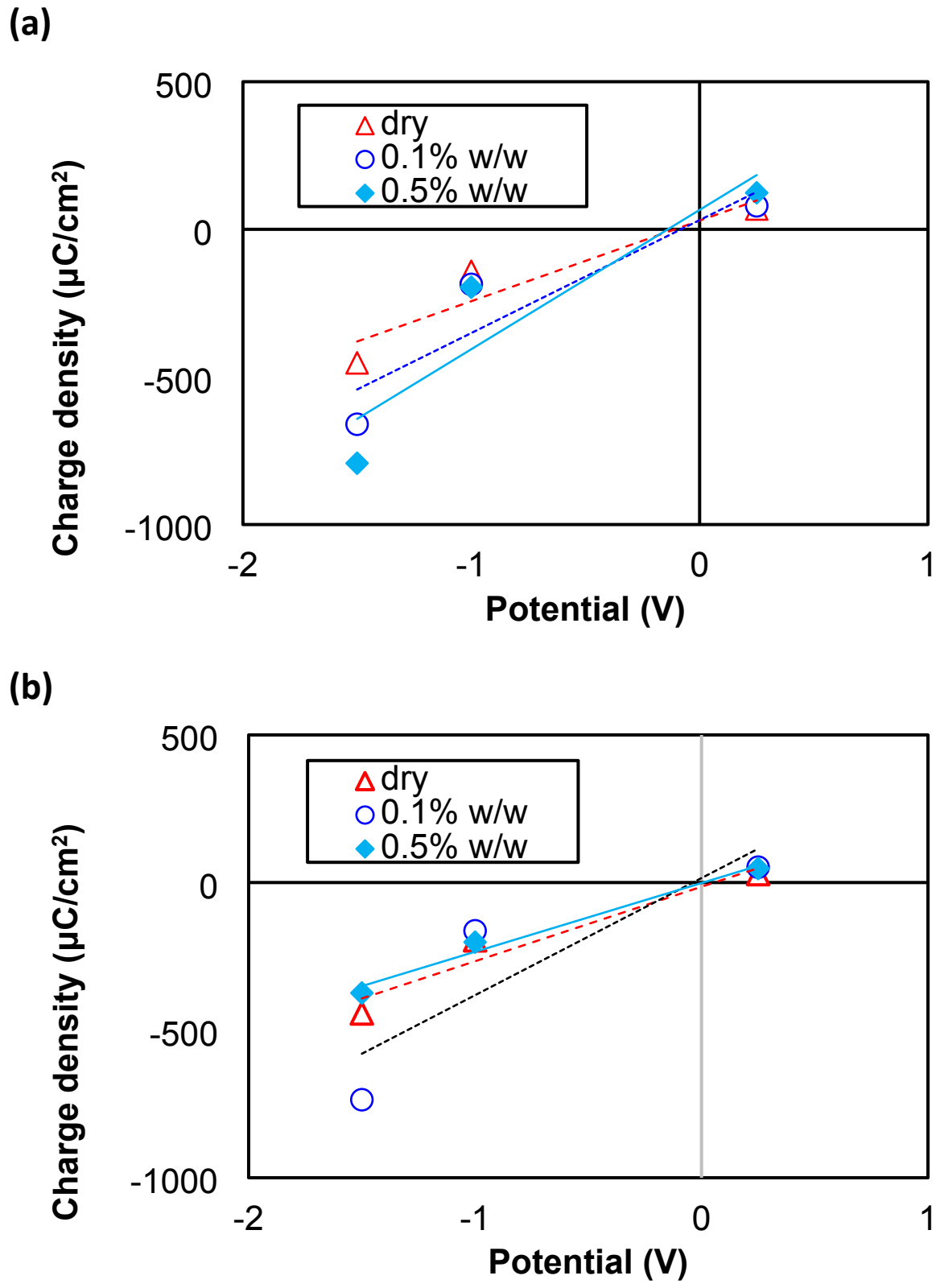

Figure S12. Surface charge densities for different concentrations of **(a)** [$C_{10}C_1$Im][BOB] and **(b)** [$C_{10}C_1$Im][BMB] in PC with added amounts of D_2O (see legend). All surface charge densities are estimated from in-situ chronoamperometry measurements during the 30 minute stabilisation period allowed after application of different applied potentials before NR measurements.

D_2O Additive Cyclic Voltammetry

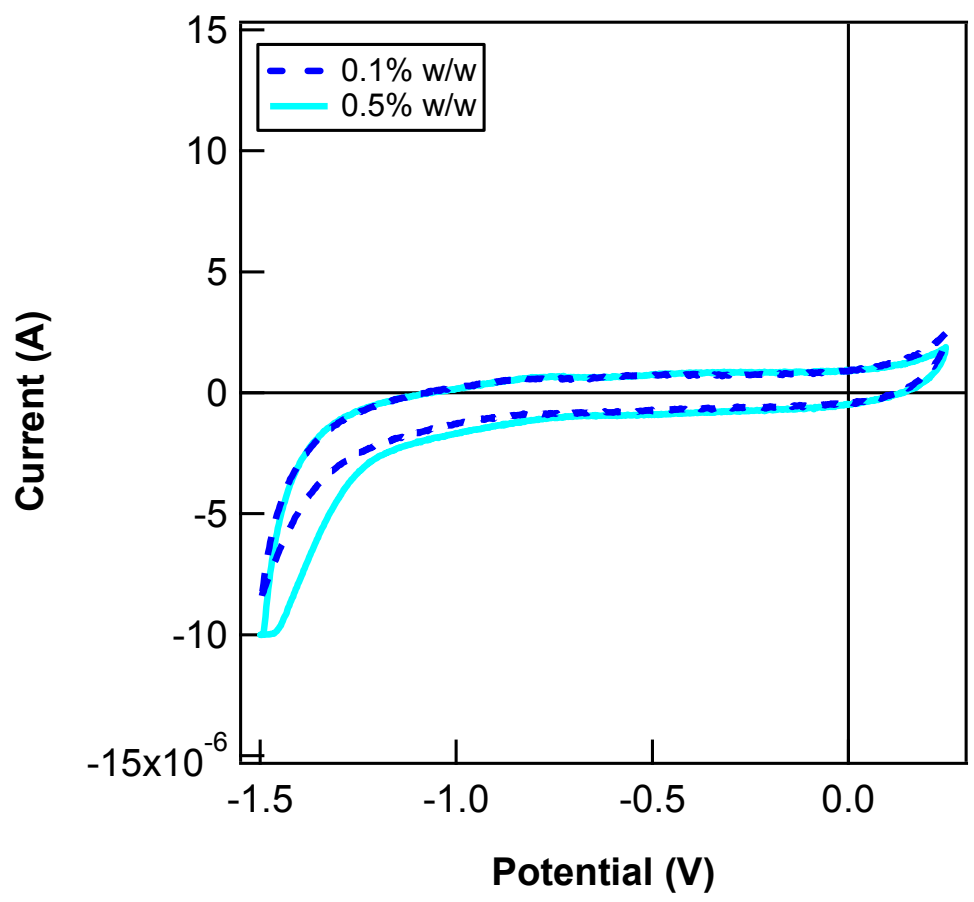

Figure S13. Cyclic voltammograms (CVs) for 20% w/w solutions of [$C_{10}C_1$Im][BMB] in PC with added amounts of D_2O. All the CVs were performed in the electrochemical NR cell at a potential sweep rate of 10 mV/s after NR measurements. The potential ranges correspond to those investigated by NR.

D_2O Additive SLD Plots

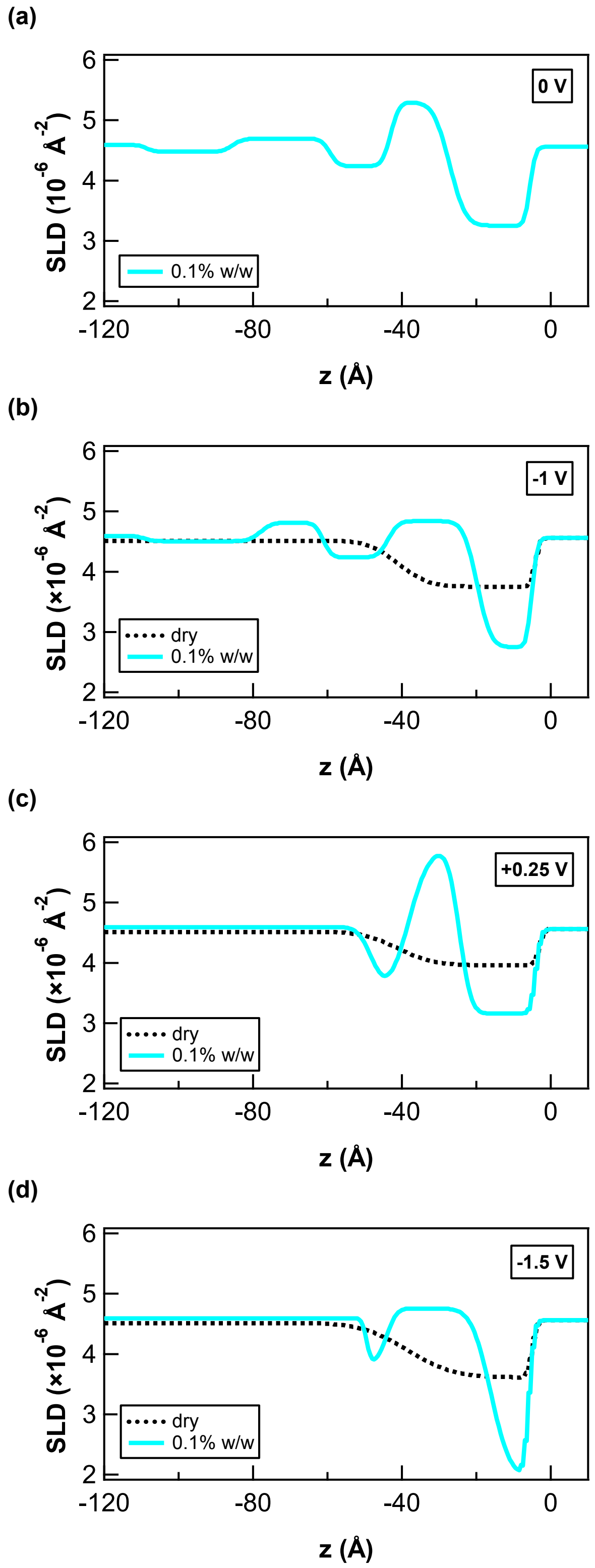

Figure S14. Effect of water on SLD profiles for 20% [$C_{10}C_1$Im][BMB] solutions SLD profiles for different applied potentials at **(a)** 0 V, **(b)** -1 V, **(c)** 0.25 V and **(d)** -1.5 V obtained from the best-fits presented, where the gold-IL interface is located at z = 0.

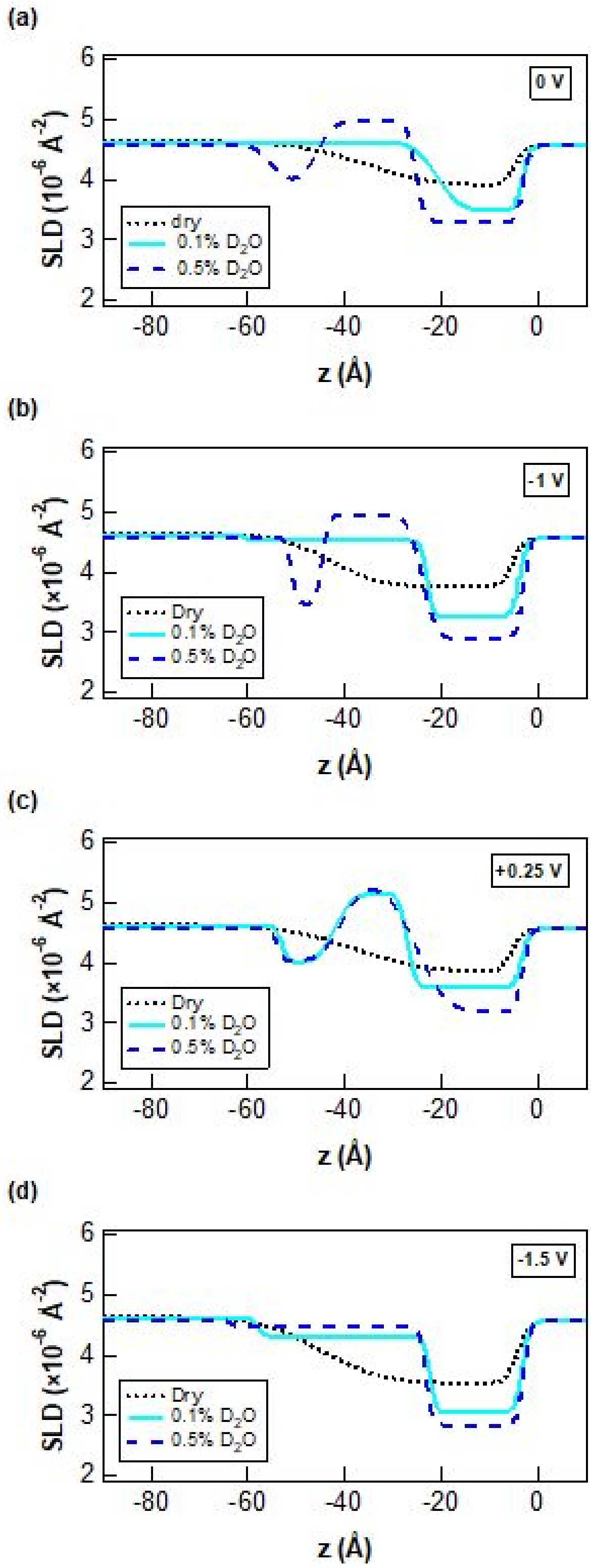

Figure S15. Effect of water on SLD profiles for 20% [$C_{10}C_1$Im][BOB] solutions SLD profiles for different applied potentials at **(a)** 0 V, **(b)** -1 V, **(c)** 0.25 V and **(d)** -1.5 V obtained from the best-fits presented, where the gold-IL interface is located at *z* =0.

D_2O Additive NR Fitting Parameters

Table S3. Best-fit parameters obtained from SLD models to NR reflectivities for 20% [$C_{10}C_1$Im][BOB] and [$C_{10}C_1$Im][BOB] in PC solutions with added concentrations of D_2O for different applied potentials at a gold electrode interface. The potentials are presented in chronological ordering of the NR experiment.

IL	D_2O conc. (% w/w)	Bulk SLD ($\times 10^6$ Å^{-2})	Potential (V)	Layer no.	Thickness (Å)	SLD ($\times 10^6$ Å^{-2})	Roughness (Å)
[$C_{10}C_1$Im][BOB]	0.1	4.62	0	1	13.1 ± 3.0	3.49 ± 0.20	3.7 ± 1.5
			-1	1	14.8 ± 1.6	3.26 ± 0.10	1.0 ± 2.0
				2	37.5 ± 14.7	4.53 ± 0.16	1.0 ± 2.0
			0.25	1	19.0 ± 6.3	3.59 ± 0.27	1.4 ± 0.2
				2	14.9 ± 5.4	5.15 ± 0.34	2.8 ± 0.7
				3	11.0 ± 5.4	4.01 ± 0.23	1.0 ± 2.0
			-1.5	1	14.9 ± 3.3	3.06 ± 0.16	1 ± 2.0
				2	35.6 ± 6.5	4.32 ± 0.08	1 ± 2.0
[$C_{10}C_1$Im][BOB]	0.5	4.59	0	1	18.3 ± 2.7	3.3 ± 0.16	1.4 ± 1.0
				2	20.3 ± 2.4	4.98 ± 0.15	2.8 ± 1.0
				3	9.3 ± 2.8	3.93 ± 0.34	3.0 ± 0.4
			-1	1	16.5 ± 1.4	2.88 ± 0.14	2.4 ± 0.4
				2	20.6 ± 2.5	4.95 ± 0.11	1.0 ± 2.0
				3	6.4 ± 2.5	3.47 ± 0.37	1.0 ± 2.0
			0.25	1	17.8 ± 1.2	3.19 ± 0.12	4.9 ± 1.9
				2	15.4 ± 2.2	5.31 ± 0.17	3.5 ± 0.7
				3	13.3 ± 2.9	4.03 ± 0.15	1.0 ± 2.0
			-1.5	1	15.8 ± 1.7	2.84 ± 0.13	1.0 ± 2.0
				2	40.7 ± 10.0	4.49 ± 0.07	1.0 ± 2.0
[$C_{10}C_1$Im][BMB]	0.1	4.59	0	1	17.2 ± 1.6	3.25 ± 0.09	3.4 ± 0.1
				2	15.8 ± 4.0	5.3 ± 0.50	1.8 ± 0.3
				3	16.4 ± 4.4	4.24 ± 0.11	1.8 ± 0.4
				4	26.2 ± 4.1	4.69 ± 0.06	2.1 ± 0.2
				5	23.0 ± 10.7	4.48 ± 0.18	1.7 ± 0.5
			-1	1	10.7 ± 1.1	2.75 ± 0.14	2.9 ± 0.3
				2	23.9 ± 1.5	4.84 ± 0.05	2.2 ± 1.2
				3	17.4 ± 1.5	4.24 ± 0.08	1.8 ± 0.3
				4	16.9 ± 3.8	4.81 ± 0.08	2.5 ± 0.2
				5	30.9 ± 5.6	4.5 ± 0.03	1.9 ± 0.9
			0.25	1	16.2 ± 2.2	3.16 ± 0.19	2.5 ± 1.5
				2	13.7 ± 2.0	5.88 ± 0.25	4.3 ± 0.2
				3	9.8 ± 4.6	3.48 ± 0.44	2.9 ± 0.2
			-1.5	1	6.0 ± 1.5	1.97 ± 0.33	4.1 ± 1.0
				2	28.0 ± 4.8	4.75 ± 0.11	2.2 ± 0.2
				3	5.3 ± 2.0	3.83 ± 0.43	1.0 ± 2.0

References

(1) Bonnaud, C.; Billard, I.; Papaiconomou, N.; Chainet, E.; Lepretre, J. C. Rationale for the implementation of reference electrodes in ionic liquids. *Physical Chemistry Chemical Physics* **2016**, *18* (11), 8148-8157, 10.1039/C5CP07652H. DOI: 10.1039/C5CP07652H.
(2) Virtanen, P.; Gommers, R.; Oliphant, T. E.; Haberland, M.; Reddy, T.; Cournapeau, D.; Burovski, E.; Peterson, P.; Weckesser, W.; Bright, J. SciPy 1.0: fundamental algorithms for scientific computing in Python. *Nature methods* **2020**, *17* (3), 261-272.
